# Modeling of the Senescence-Associated Phenotype in Human Skin Fibroblasts

**DOI:** 10.3390/ijms23137124

**Published:** 2022-06-27

**Authors:** Marta Gerasymchuk, Gregory Ian Robinson, Olga Kovalchuk, Igor Kovalchuk

**Affiliations:** Department of Biological Sciences, University of Lethbridge, Lethbridge, AB T1K 3M4, Canada; marta.gerasymchuk@uleth.ca (M.G.); g.robinson@uleth.ca (G.I.R.)

**Keywords:** aging, skin, fibroblast, hydrogen peroxide, stress-induced premature senescence

## Abstract

Modern understanding of aging is based on the accumulation of cellular damage during one’s life span due to the gradual deterioration of regenerative mechanisms in response to the continuous effect of stress, lifestyle, and environmental factors, followed by increased morbidity and mortality. Simultaneously, the number of senescent cells accumulate exponentially as organisms age. Cell culture models are valuable tools to investigate the mechanisms of aging by inducing cellular senescence in stress-induced premature senescence (SIPS) models. Here, we explain the three-step and one-step H_2_O_2_-induced senescence models of SIPS designed and reproduced on different human dermal fibroblast cell lines (CCD-1064Sk, CCD-1135Sk, and BJ-5ta). In both SIPS models, it was evident that the fibroblasts developed similar aging characteristics as cells with replicative senescence. Among the most noticeable senescent biomarkers were increased β-Gal expression, high levels of the p21 protein, altered levels of cell-cycle regulators (i.e., CDK2 and c-Jun), compromised extracellular matrix (ECM) composition, reduced cellular viability, and delayed wound healing properties. Based on the significant increase in senescence biomarkers in fibroblast cultures, reduced functional activity, and metabolic dysfunction, the one-step senescence model was chosen as a feasible and reliable method for future testing of anti-aging compounds.

## 1. Introduction

Aging can be viewed as the accumulation of consecutive changes over time in response to stress, lifestyle, and environmental factors, ultimately causing irreparable damage and maladaptation in the function of cells, ECM, cell communication, intercellular signaling, and leads to age-related diseases and death [1,2]. Cutaneous aging is a complex phenomenon involving two simultaneously occurring processes: an intrinsic one, known as chronological aging, which is genetically determined, and extrinsic aging, which is due to environmental factors such as chronic sun exposure, known as photoaging [3]. It is manifested by the gradual process of wrinkle development, skin sagging, and drooping. The naturally (intrinsic) aged skin looks dry, and has fine wrinkles, but is still smooth and light [4]. In contrast, the extrinsically photo-aged skin has thick layers (“leathery aspect”) and rough wrinkles with pigmentation (“age-spots”, which are actinic lentigines) and capillary telangiectasia [5,6,7,8].

There are several biomarkers of senescence that could be detected in all somatic cells. The most well-known among them are: (i) senescence-associated β-galactosidase (β-Gal) activity, (ii) the overexpression of cell cycle arrest proteins such as p16, p21, p53, (iii) the depletion of mitochondrial DNA (mtDNA), (iv) changes in the expression of senescence-associated microRNAs, (v) telomere attrition, (vi) and decreased expression of Ki-67 protein, which is associated with cell proliferation [7,9,10,11,12].

Among all cutaneous layers, the dermis is the target source for current anti-aging and rejuvenation therapies. This dermal layer is rich in fibroblasts which produce ECM or skin scaffold, which mainly consists of collagen, elastin, and hyaluronic acid [13]. Older skin demonstrates exhaustion of all these ECM components. Age-related increases in matrix metalloproteinases (MMPs) production are directly responsible for collagen degradation leading to a decline in collagen content at about 2% per year [14,15]. Dermal fibroblasts experience an alteration in the equilibrium between the synthesis of collagen and the synthesis of collagen-degrading enzymes under the influence of various stressors throughout life with further enhancement of dermal structural impairment [16]. Moreover, increased accumulation of degraded collagen fibers work as entrapment that manifests itself in the reduction of fibroblast functional quality and quantity; likewise, a decline in collagen and other ECM proteins renewal capacity is observed with age [15]. This is why a detailed analysis of fibroblast function is important to understand the pathogenesis of dermal aging.

Consequently, cultured human diploid fibroblasts (HDF) have become popular in vitro cell systems in types of research designed to study conditions associated with alterations in replicative potentials, such as cancer or senescence. The limited replicative ability of HDF makes them a suitable model for cellular aging and potentially for rejuvenation studies. Moreover, senescent fibroblasts exert distinct morphology, metabolic dysfunction, and diminished functional qualities.

Present-day studies in the field of biogerontology interchangeably use the terms cell senescence, cellular aging, and replicative senescence for utilizing normal diploid cells in culture, which undergo a multitude of changes during serial subcultivation resulting in the permanent termination of cell division known as the Hayflick limit [17]. In vitro studies of age-related changes in the physiology, biochemistry, mechanobiology, and molecular biology of cultured cells have considerably boosted the understanding of the fundamental principles of cellular senescence. Numerous in vitro models have been proposed to explain the aging process via cellular senescence, but none of them appear to be fully comprehensive.

In 1961, Hayflick and Moorhead described the first model based on serial passaging of fibroblasts in culture that was considered as the process of cellular aging; it resulted in the end-stage irreversible growth arrest in G1/G0, known as replicative senescence (RS). In this state, cells are alive, exhibit an altered metabolic activity, and generally resist undergoing apoptosis [17,18]. Thus, these cells are in vitro senescent aged cells and are one of the most widely used cellular aging models [19]. Thus, induction of RS is a continuous long-term process of cell passaging that might take anywhere from a few months up to a year, or even longer, and require expensive maintenance. Considering this, most research groups are looking into models of cellular senescence that does not require waiting for the replicative limit to be reached.

At the end of the last century, Toussaint and Remarcle (1995) presented research data showing that cellular aging can be accelerated by using a wide range of non-lethal stressors and proposed the term stress-induced premature senescence (SIPS) typically based on the principles of oxidative stress [20,21,22]. In accordance with multiple experimental data and theoretical studies, fibroblasts, keratinocytes, melanocytes, or umbilical vascular endothelial cells were exposed to chronic and acute oxidative stress protocols, including sublethal stresses such as UV, hyperoxia, hypoxia, hydrogen peroxide, ethanol, bleomycin, mitomycin C, etc., [19,22,23].

Most of the experimental setups for SIPS models depend on the cell type and are modulated via single or multiple applications of designated stressors for diverse periods of time in subcytotoxic doses. Advantages of those models were highlighted by high feasibility, low cellular recovery, fast exhaustion of the replicative potential, and eventually irreversible growth arrest that allowed reduced time and cost maintenance. Among commonly used SIPS in vitro models are: (a) ultraviolet light exposure [5,6,7,9,24,25,26,27,28,29]; (b) X-ray and γ-irradiation [30,31,32,33,34]; (c) mitomycin C, bleomycin, or actinomycin D-induced SIPS [35,36,37]; (d) hydrogen peroxide (H_2_O_2_) [38,39,40,41,42]. Albeit the stressors mentioned above have many advantages, none of them is optimal to reflect all mechanisms involved in cellular senescence. At the same time, oxidative stress induced by H_2_O_2_ is one of the most popular and reproducible models of cellular senescence. H_2_O_2_ causes DNA strand breaks and activates poly (ADP-ribose) polymerase, leading to depletion of the cellular pool of NAD+ and ATP [43,44]. The excessive amount of reactive oxygen species (ROS) produced from H_2_O_2_ damages the cellular membrane, decreases antioxidant activity, and more vigorously potentiates endogenous free radical generation. Induced changes corresponded to previously mentioned typical morphological, biomolecular, and genetic alterations found in SIPS.

The effectiveness of the H_2_O_2_-induced premature senescence model depends on the cell-line type, maintaining procedures, dose, time of exposure, and type of solvent (Table 1). To date, there is no unique protocol that can cover all the aspects and needs related to cell culture and supplements which is why research groups use different variations of H_2_O_2_ exposure to establish an efficient senescence model.

In this study, we examined different models of H_2_O_2_-induced SIPS to compare the efficacy and repeatability of SIPS in normal human fibroblasts cell lines CCD-1064Sk, CCD-1135Sk, and BJ-5ta. CCD-1064Sk and CCD-1135Sk cell lines represent healthy human dermal fibroblasts that originate from neonatal foreskin and adult skin, respectively. BJ-5ta fibroblasts originate from neonatal foreskin that have been immortalized using human telomerase reverse transcriptase (hTERT). Utilizing these three cell lines, we compared age-induced and stressor-induced senescence of neonatal foreskin fibroblasts and adult human dermal fibroblasts, as well as analyzed senescence that is not associated with telomere degradation.

## 2. Results

### 2.1. Setup of the Senescence Model System

**Figure 1:**
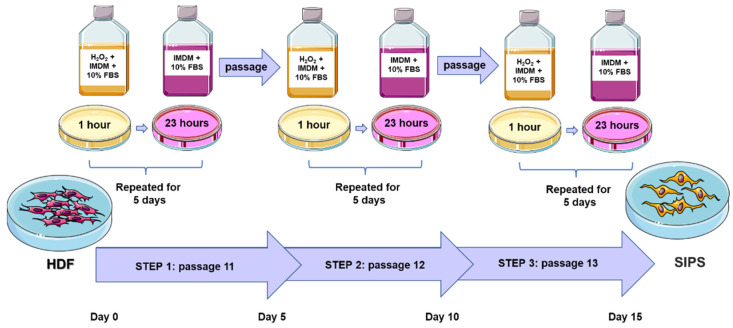
Three-step model of hydrogen peroxide stimulated premature cellular senescence. Cultivated cells were treated daily with H_2_O_2_ for one hour over a five-day period in each step. Each step started when cell confluency was approximately 70%. HDF, human dermal fibroblasts; SIPS, stress-induced premature senescence. This figure was created using images from Servier Medical Art Commons Attribution 3.0 Unported License (http://smart.servier.com accessed on 26 September 2020).

To understand age-dependent changes that occur in the skin, we designed and tested two different models of senescence using dermal fibroblasts. First, normal human fibroblasts cell lines CCD-1064Sk, CCD-1135Sk, and BJ-5ta were treated with H_2_O_2_ at various concentrations to find a line that would recapitulate the development of SIPS without direct lethal effect. Thus, in the three-step senescence model, dermal fibroblasts were consistently exposed to the 25 µM, 50 µM, 100 µM, 150 µM, 200 µM, 250 µM, 300 µM, and 350 µM of H_2_O_2_ for 15 days divided into three five-day-steps (Figure 1). H_2_O_2_ treatment solution was prepared in IMDM cell culture medium with 10% FBS that gave additional nutrients to the cells and decreased the harmful effect of higher doses of H_2_O_2_. SIPS fibroblasts in culture normally developed very slowly, which increases the cost of studies and complicates the consistency of results after repetition of experiments (data not shown). However, the three-step senescence model that we developed showed robust SIPS changes in different dermal fibroblast cell lines.

**Figure 2:**
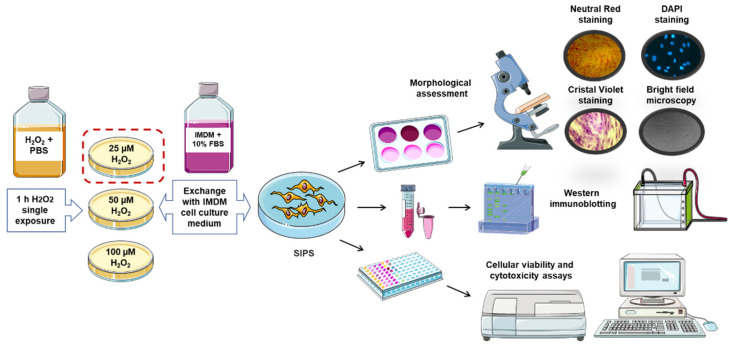
One-step model of hydrogen peroxide stimulated premature cellular senescence. SIPS, stress-induced premature senescence. Cultivated cells were treated with H_2_O_2_ dissolved in PBS for one hour after 70% cell confluency was achieved. This figure was created using images from Servier Medical Art Commons Attribution 3.0 Unported License (http://smart.servier.com accessed on 26 September 2020).

The second senescence model focused on the fast induction of SIPS in skin fibroblasts (CCD-1064Sk and BJ-5ta cell lines). In this model, we also exposed cells to various concentrations of H_2_O_2_ from 25 µM to 350 µM; however, fibroblasts were treated once with H_2_O_2_ for one hour (Figure 2). Since fibroblasts are only treated with H_2_O_2_ once, this method is called a one-step model. In addition, H_2_O_2_ was dissolved in the PBS to eliminate the protective effect of the cell culture medium with FBS supplementation and induce a stress-like calorie restriction condition. PBS did not show adverse effects on cell cultures within one hour (data not shown) and was used in the subsequent experiments as a solvent for H_2_O_2_. We will compare these two senescence models and the effects of various concentrations of H_2_O_2_ in the SIPS developmental model.

### 2.2. Senescence-Induced Morphological Transition

Normal human fibroblasts have a limited in vitro lifespan when cells enter a non-dividing, senescent state, known as the Hayflick limit, which is one of the markers of senescence [45,46]. Neonatal foreskin CCD-1064Sk fibroblasts have approximately 54 population doubling level (PDL) that usually corresponds to cultured passage 25–28 for this specific cell line, while the rate of population doubling is three days. For normal human adult skin fibroblasts CCD-1135Sk, PDL ≥ 46 (15–20 passages) while the rate of population doubling is four days. In contrast, human foreskin normal BJ-5ta hTERT-immortalized cell line (CRL-4001™) was TERT-immortalized at PDL of 58 and doubles every two days. Cell stocks obtained from ATCC were subcultured to reach three different passages depending on the type of cell line. To determine the senescence status of those cells, first we monitored their PDL, evaluated morphological changes, and senescence ratios as by β-Gal assay [47]. We then tested molecular senescence biomarkers and functional capabilities of the aged cells.

Phase-contrast imaging was used to characterize senescent dermal fibroblasts (Figure S1). While early fibroblast passages formed dense cultures and had small, elongated cell bodies, late passages displayed shape changes with large, flat cellular morphology, whereby cell and nuclear features looked nearly transparent. In addition, another critical aspect of passaging was determined—the loss of replicative capacity. We observed RS in prolonged culture fibroblasts (Figure S2). Like late passage fibroblasts, prematurely aged fibroblasts treated with H_2_O_2_ showed a substantial reduction in growth speed and developed characteristics similar to other senescence models designed in our laboratory, albeit, they still demonstrated some replicative ability.

To find the optimal concentration of H_2_O_2_ for SIPS induction and to reduce the number of H_2_O_2_ treatments, we took images of H_2_O_2_-treated fibroblasts for the one-step model with and without staining procedures (Figure S3). The morphological results of one-step senescence models showed 100 µM of H_2_O_2_ affected cells perniciously. H_2_O_2_ concentrations of 50 µM and 100 µM showed a progressive detrimental impact on cell viability and number. At the same time, 25 µM of H_2_O_2_ showed a significant reduction in fibroblasts viability and replicative ability.

Abnormalities in nuclear morphology and lysosomal dysfunction are two well-known characteristics of senescence [48,49]. Crystal violet stains DNA and proteins and was used to visualize nuclei and cell structure, while neutral red is absorbed by lysosomes and was performed to see if altered lysosomal uptake was occurring which would suggest lysosomal dysfunction. Representative images show qualitative differences between controls and hydrogen peroxide treatments. All hydrogen peroxide treatments appeared to have decreased crystal violet staining with altered nuclear/cell morphology (Figure S3B,E,H,K). Changes in lysosome morphology was present with noticeable reduction in neutral red uptake as concentration of hydrogen peroxide is increased (Figure S3C,F,I,L).

### 2.3. β-Galactosidase as a Biomarker for Senescence-Associated Phenotype

**Figure 3:**
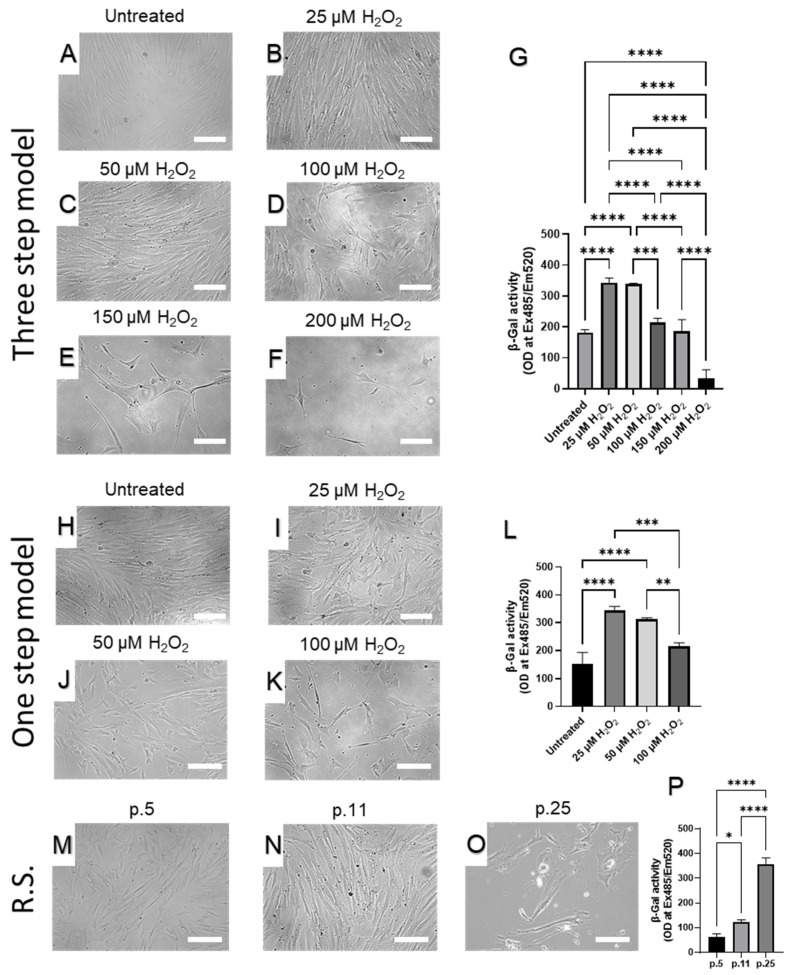
Human skin fibroblasts (CCD-1064Sk) p.13 exposed to H_2_O_2_ (Step 3 of the three-step senescence model). (**A**–**F**) The images represent gradual changes in cell quantity and quality after 15 days (three-step model) of exposure to different concentrations of H_2_O_2_: (**A**) Untreated; (**B**) 25 µM; (**C**) 50 µM; (**D**) 100 µM; E, 150 µM; F, 200 µM; (**G**) Levels of β-Gal activity after H_2_O_2_ exposure on day five, step 3 (three-step model), were compared with untreated cells (CCD-1064, p.13). (**H**–**K**), the images represent gradual changes in cell quantity and quality after five days (one-step model) of exposure to different concentrations of H_2_O_2_: (**H**) untreated; (**I**) 25 µM; (**J**) 50 µM; (**K**) 100 µM; (**L**) Levels of β-Gal activity after H_2_O_2_ exposure on day five (one-step model of senescence). (**M**–**O**), The images represent gradual changes in cell quantity and quality in different passages (p.5, p.11, and p.25 respectively) of CCD-1064Sk skin fibroblasts during replicative senescence (RS) development; (**P**) Levels of β-Gal activity in different passages of dermal fibroblasts (CCD-1064Sk). Data were analyzed with a one-way ANOVA test (Tukey post-hoc multiple comparison test). Significance is indicated within the figures using the following scale: *, *p* < 0.05; **, *p* < 0.01; ***, *p* < 0.001; ****, *p* < 0.0001. Bars represent mean ± SD. Scale bars = 100 µm.

To determine the extent of senescence in the dermal fibroblast cultures during serial passaging or the proposed senescence-induced models, we measured the senescence-associated β-galactosidase (β-Gal) levels (Figure 3), which is commonly used as a biomarker for senescence [47,50,51].

The three-step senescence model demonstrated a progressive decline in fibroblast number (Figure 3A–F) and conversely, a significant increase in β-Gal levels (Figure 3G) in both 25 and 50 µM of H_2_O_2_ compared to the control. Importantly, we noticed a relative decline in β-Gal levels in fibroblasts treated with 100 µM and 200 µM of H_2_O_2_. This was likely due to a decrease in the number of fibroblasts at the end of step three of the three-step senescence model. Due to the limited number of cells present at the end of three-step model that were treated with 250 µM, 300 µM, and 350 µM of H_2_O_2_, β-Gal measurements were unreliable or undetectable (data not shown).

In the one-step SIPS, similar to the three-step model, we noticed H_2_O_2_ dose-dependent gradual reduction CCD-1064sk fibroblasts quantity (Figure 3H–K). The β-Gal levels of were also significantly increased in the prematurely aged fibroblasts during the one-step model of senescence (Figure 3L). Additionally, we noticed a relative decline in β-Gal levels in fibroblasts treated with 100 µM of H_2_O_2._ This was likely due to a substantial shortage in the number of fibroblasts at the end of day 5 of the one-step SIPS model.

Besides morphological and quantity changes in CCD-1064Sk fibroblasts associated with increased passage number (Figure 3M–O), we detected that compared to the early passage cultures, the β-Gal levels in late passages were significantly higher (Figure 3P).

### 2.4. Senescence-Associated Changes in Nuclear Morphology

Morphologically, young fibroblasts were identified as elongated cells exhibiting a spindle-like shape that adhered to plates (Figure S2A). Senescent fibroblasts and those exposed to H_2_O_2_, demonstrated cellular size enlargement and irregular shape, while appearing to be reduced in cell number (Figures S2C and S3). Findings obtained during microscopy examinations of cell cultures with and without staining motivated us to investigate whether H_2_O_2_ exposure influenced nuclear architecture. Therefore, fibroblasts were stained with DAPI, a fluorescent stain that binds strongly to adenine–thymine-rich regions in DNA.

**Figure 4:**
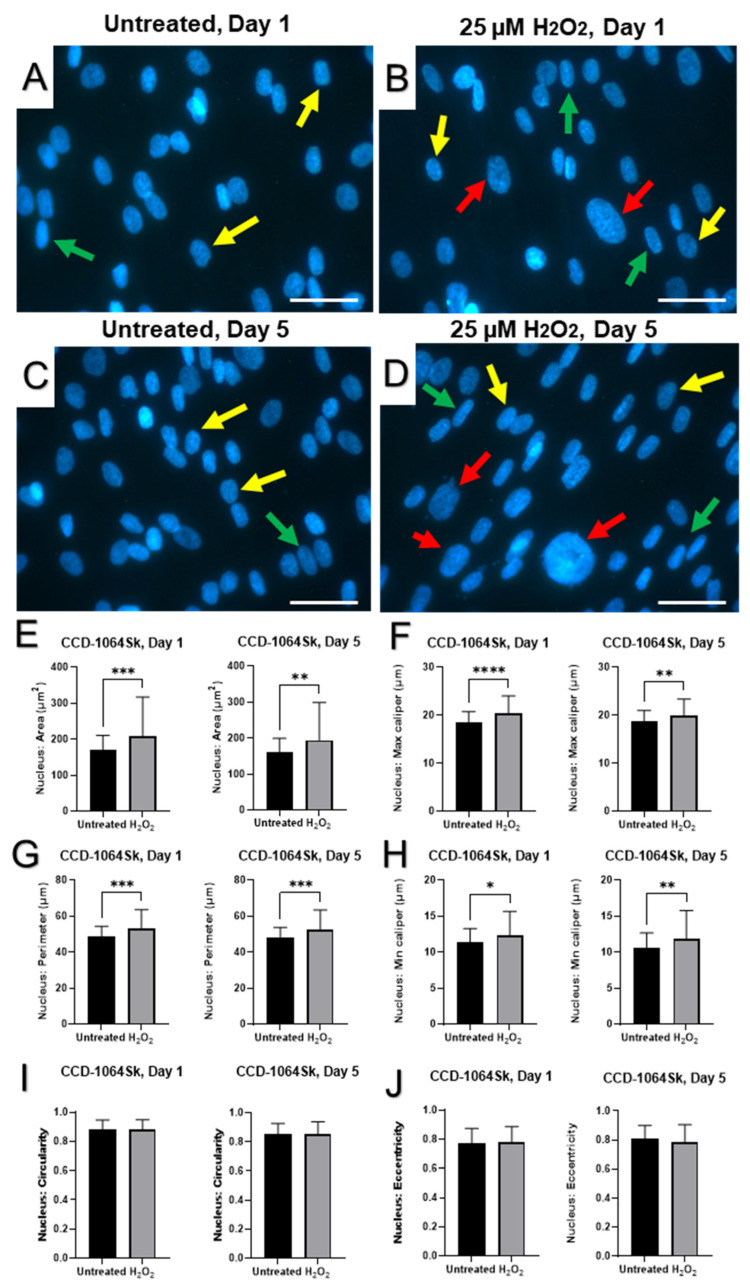
DAPI-stained nuclei of dermal fibroblasts (CCD-1064Sk), p.11 (one-step model). Pictures A-D represent nuclear changes observed by immunofluorescence microscopy on days 1 and 5 after a single 25 µM H_2_O_2_ exposure. Arrows point to nuclei with specific nuclear shapes: yellow—round, green—elongated, and red—gigantic/irregular. (**A**) Untreated cells on day 1; (**B**) prematurely aged fibroblasts after 25 µM of H_2_O_2_ exposure on day 1; (**C**) untreated cells on day 5; (**D**) senescent fibroblasts on day 5. Nuclear parameters measured utilizing QuPath in each sample: (**E**) Nuclear area; (**F**) Nuclear max caliper; (**G**) Nuclear perimeter; (**H**) Nuclear min caliper; (**I**) Nuclear Circularity; (**J**) Nuclear eccentricity. Data were analyzed with a Student’s unpaired *t*-test for each separate parameter. Significance is indicated within the figures using the following scale, * *p* < 0.05; **, *p* < 0.01; ***, *p* < 0.001; ****, *p* < 0.0001. Bars represent mean ± SD. Scale bars = 20 µm.

**Figure 5:**
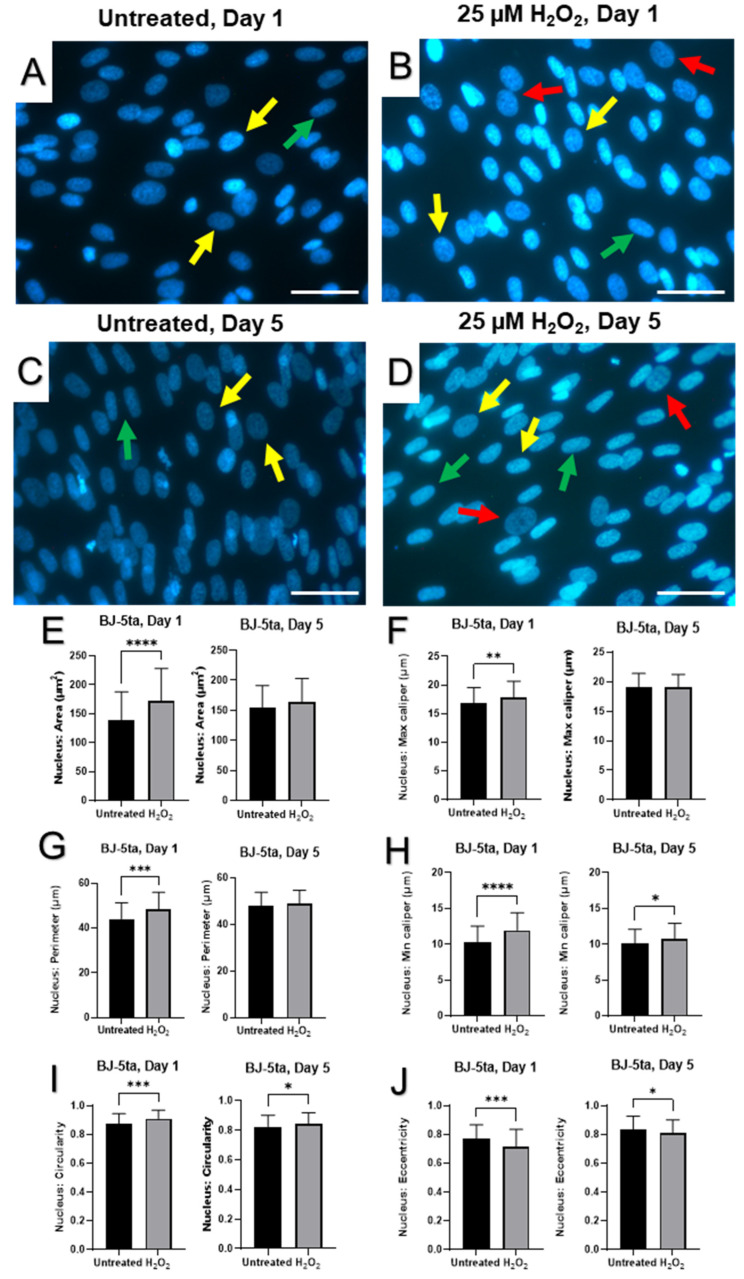
DAPI-stained nuclei of dermal fibroblasts (BJ-5ta), p.42 (one-step model). Pictures (**A**–**D**) represent nuclear changes observed by immunofluorescence microscopy on days 1 and 5 after a single 25 µM of H_2_O_2_ exposure. Arrows depict changes in nuclear shapes: yellow—round, green—elongated, and red—gigantic/irregular. (**A**) Untreated cells on day 1; (**B**) prematurely aged fibroblasts after 25 µM of H_2_O_2_ exposure on day 1; (**C**) untreated cells on day 5; (**D**) senescent fibroblasts on day 5. Nuclear parameters measured utilizing QuPath in each sample: (**E**) nuclear area; (**F**) nuclear max caliper; (**G**) nuclear perimeter; (**H**) nuclear min caliper; (**I**) nuclear circularity; (**J**) nuclear eccentricity. Data were analyzed with a Student’s unpaired *t*-test for each separate parameter. Significance is indicated within the figures using the following scale: *, *p* < 0.05; **, *p* < 0.01; ***, *p* < 0.001; ****, *p* < 0.0001. Bars represent mean ± SD. Scale bars = 20 µm.

Nuclear DAPI staining showed significant enlargement and variability in size and shape of nuclei in aged fibroblasts compared to healthy cells (Figure 4 and Figure 5). Although some nuclei were elongated in the untreated conditions, an apparent increase in prevalence and severity of elongation of fibroblast nuclei were seen in CCD-1064Sk cell line exposed to H_2_O_2_ for five days (Figure 4A,D). Although many nuclei were elongated in the CCD-1064Sk fibroblasts, some cells retained a round nuclei architecture (Figure 4D). In addition, we noticed the appearance of gigantic nuclei with irregular shapes in the CCD-1064Sk cell line when treated with H_2_O_2_ (Figure 4B,D). In contrast, prematurely senescent BJ-5ta fibroblasts exhibited predominantly round and irregularly sized nuclei on day one with subsequent transformation into an elongated morphology by day five (Figure 5B,D). Other nuclear parameters, such as perimeter, max caliper, and min caliper as well as the nuclear area, significantly increased in CCD-1064Sk senescent fibroblast (Figure 4E–H). A similar trend with more minor differences was found in BJ-5ta fibroblasts (Figure 5). In contrast, nuclear circularity and eccentricity remained almost unchanged between untreated and SIPS CCD-1064Sk fibroblasts (Figure 4I,J). In BJ-5ta fibroblasts, a significant elevation in nuclear circularity and decreased eccentricity was found in prematurely aged fibroblasts (Figure 5I,J). Overall, both cell lines had significantly altered nuclei architecture when treated with 25 µM of H_2_O_2_ for five days, suggesting nuclear envelope dysfunction [48].

### 2.5. Senescence-Related Changes to Gene Expression Profiles Are Accompanied by Elevation in the Expression of Cell-Cycle Regulators

Based on morphological studies, we hypothesized expression of cell cycle regulators was modulated and would lead to functional deterioration and metabolic dysfunction. Hence, it was decided to test the protein expression levels of p16, p21, and p53, cell-cycle progression regulators, senescence-associated markers in CCD-1135Sk, CCD-1064Sk, and BJ-5ta cultures. This analysis would also show the reliability of our cellular senescence model.

**Figure 6:**
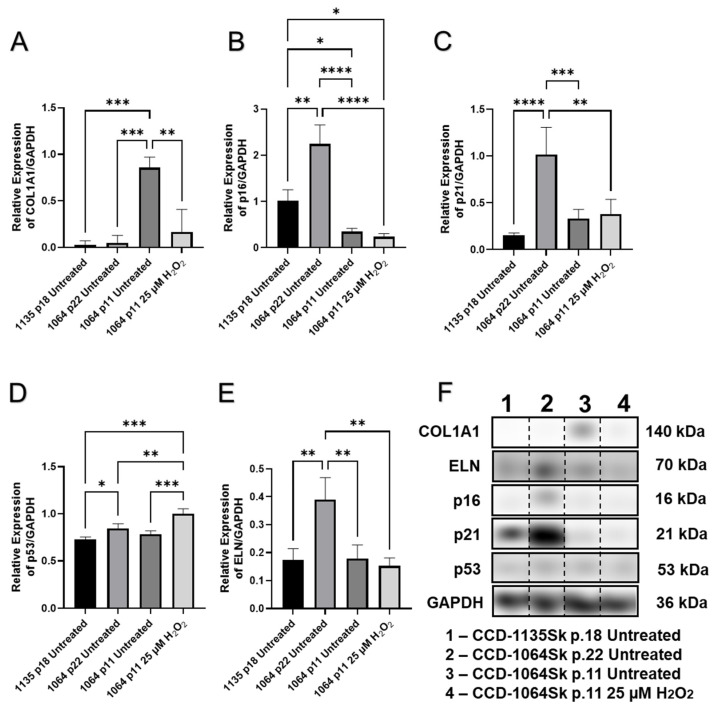
The expression of cellular checkpoint regulators and collagen in aged fibroblasts from different cell lines. Fibroblast’s cell-lines CCD-1135Sk (p.18) and CCD-1064Sk (p.22) aged naturally by replicative senescence, and CCD-1064Sk (p.11) was aged by the one-step H_2_O_2_ senescence model. Figures (A–E) show protein expression levels for selected genes measured by Western blot. (**A**) COL1A1 expression; (**B**) p16 expression; (**C**) p21 expression; (**D**) p53 expression; (**E**) elastin expression; (**F**) levels of protein expression detected in Western blots. Glyceraldehyde-3-phosphate dehydrogenase (GAPDH) was used as a loading control. Relative densitometry was presented as a ratio of target protein to GAPDH. Data were analyzed with a one-way ANOVA test (Tukey post-hoc multiple comparison test). Significance (p) is indicated within the figures using the following scale: *, *p* < 0.05; **, *p* < 0.01; ***, *p* < 0.001; ****, *p* < 0.0001. Bars represent mean ± SD. The samples for each protein were run on the same gel (Figures S4–S9). Since the samples were not run consecutively on the same gel, they were spliced for presentation purposes only. No image enhancements were applied.

**Figure 7:**
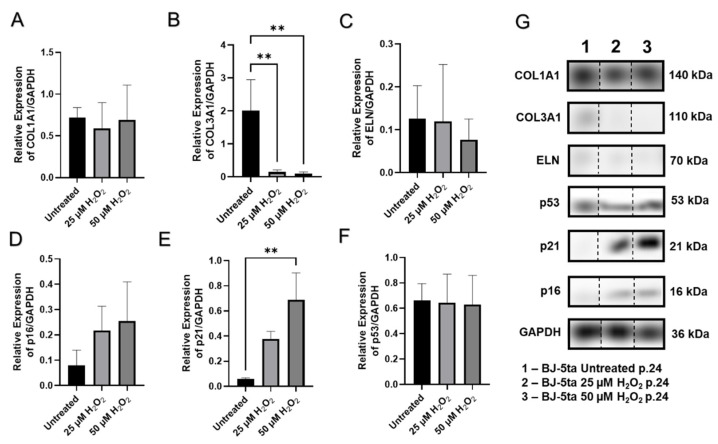
The effects of age on cell cycle checkpoint regulators in BJ-5ta (p.24) fibroblasts. Western blots showing protein levels of COL1A1 (**A**), COL3A1 (**B**), ELN (**C**), p16 (**D**), p21 (**E**), and p53 (**F**). Relative densitometry was presented as a ratio of target protein to GAPDH (**G**). Data were analyzed with a one-way ANOVA test (Tukey post-hoc multiple comparison test). Significance (p) is indicated within the figures using the following scale: **, *p* < 0.01. Bars represent mean ± SD. Original Western blot images were included in Figures S10–S16. The samples for each protein were run on the same gel (Figure S10–S16). Since the samples were not run consecutively on the same gel, they were spliced for presentation purposes only. No image enhancements were applied.

The Western blot image shown in Figure 6 indicates that the p16 protein levels were increased in the CCD-1064Sk fibroblast cells with RS (CCD-1064Sk, p.22) compared to both untreated healthy fibroblasts (CCD-1064Sk, p.11), and surprisingly, the 25 µM of H_2_O_2_-exposed fibroblasts (CCD-1064Sk p.11, 25 µM of H_2_O_2_). Importantly, our 25 µM H_2_O_2_-induced SIPS model (CCD-1064Sk p.11, 25 µM of H_2_O_2_) did not show a significant difference in p16 expression from the control (CCD-1064Sk, p.11, untreated) as seen in Figure 6B; however, in Figure 7D, at higher doses of H_2_O_2_, p16 is appears to be upregulated, likely indicating there is insufficient statistical power to observe small increases in expression. p16 modulates the growth inhibitory state by keeping retinoblastoma protein (Rb) hypophosphorylated via targeting CDK4 and CDK6 complexes, ensuing repression of E2F target genes required for S-phase onset. Similar repressive effects of E2F target genes are achieved by cyclin-dependent kinase inhibitor p21, which inhibits the action of CDK2 activity arresting the cell cycle in G1. The analysis of p21 expression levels indicates that p21 expression was significantly higher in replicative senescence CCD-1064Sk p.22 fibroblasts compared to untreated CCD-1135Sk p.18 fibroblasts, untreated CCD-1064Sk p.11 fibroblasts, and CCD-1064Sk p.11 fibroblasts that were exposed to hydrogen peroxide (Figure 6C). The expression of the p53 protein was elevated in CCD-1064Sk p.22 and 25 µM H_2_O_2_-treated fibroblasts compared with CCD-1135Sk p.18 and untreated fibroblast (Figure 6D).

Western blot analysis of BJ-5ta foreskin fibroblasts showed a rise in p16 and p21 expression levels in a dose-dependent manner (Figure 7D, and Figure 7E, respectively). Interestingly, the p53 protein levels did not show significant differences (Figure 7F).

**Figure 8:**
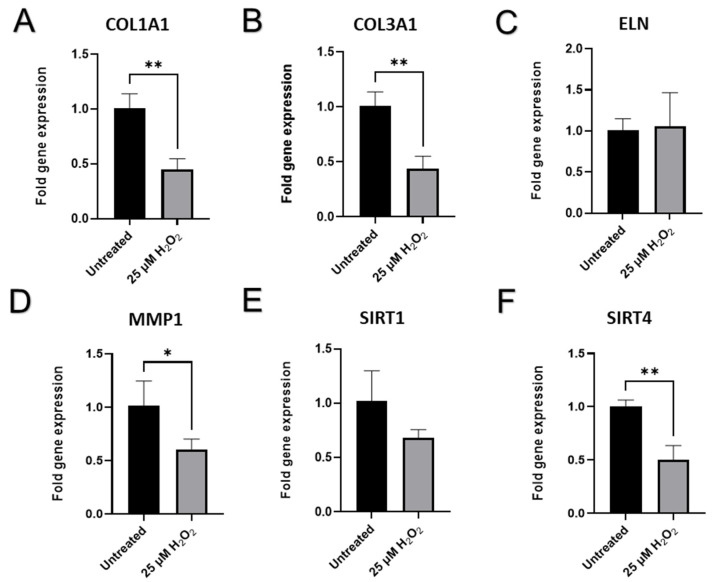
The effects of age on mRNA production of extracellular matrix components and functional regulators in BJ-5ta (p.24) fibroblasts. Changes of mRNA expression as measured by RT-qPCR for (**A**) *COL1A1*; (**B**) *COL3A1*; (**C**) *ELN; (***D**) *MMP-1*; (**E**), *SIRT-1*; (**F**) *SIRT-4*. Data were with an unpaired Student’s *t*-test. Significance (p) is indicated within the figures using the following scale: *, *p* < 0.05; **, *p* < 0.01. Bars represent mean ± SD. Additional non-significant mRNA expression of genes can be seen in Figure S25.

One of the typical features of SIPS is lost functional activity. It is well-known that fibroblasts are the main generators of extracellular matrix (ECM) components such as elastin and various types of collagens. Western blots showed significant reduction in type I collagen (COL1A1) expression in senescent CCD-1135Sk p.18, CCD-1064Sk p.22 and aged CCD-1064Sk p.11 fibroblasts compared to the untreated CCD-1064Sk controls (Figure 6A,F). The mRNA expression level of type III collagen (*COL3A1*) showed a similar tendency as *COL1A1* (Figure 8A,B) in BJ-5ta fibroblasts with protein levels significantly reduced in both hydrogen peroxide treatment groups compared to the control (Figure 7B).

Another key protein of the ECM is elastin, encoded by the *ELN* gene. It maintains elasticity and helps cells to regain their shape after stretching or contracting. Unexpectedly, increased expression of elastin protein was observed in senescence fibroblasts CCD-1064Sk (p.22) compared to all treatments (*p* < 0.01, Figure 6E,F).

**Figure 9:**
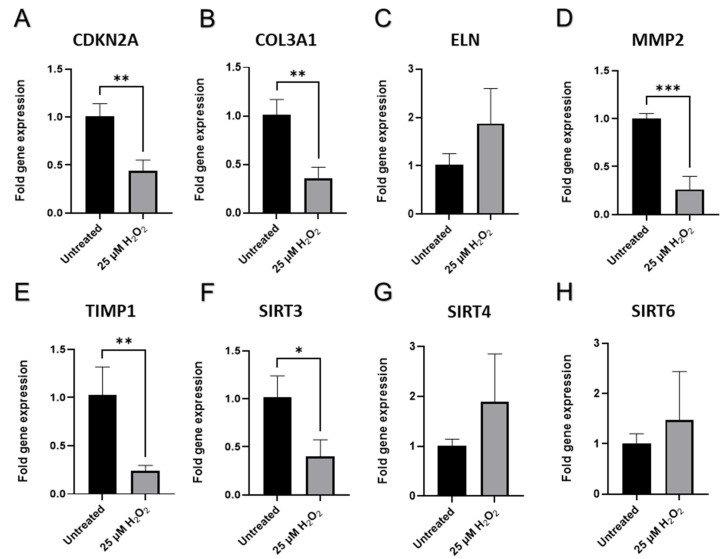
Senescence-associated gene expression in CCD-1064Sk (p.11) dermal fibroblasts (one-step senescence model). Changes in mRNA expression levels for selected genes measured by RT-qPCR in CCD-1064Sk cells: (**A**) *CDKN2A*; (**B**) *COL3A1*; (**C**) *ELN*; (**D**) *MMP2*; (**E**) *TIMP1*; (**F**) *SIRT3*; (**G**) *SIRT4*; (**H**) *SIRT6*. Data were analyzed with an unpaired Student’s *t*-test. Significance is indicated within the figures using the following scale: ns, *, *p* < 0.05; **, *p* < 0.01; ***, *p* < 0.001. Bars represent mean ± SD.

Next, mRNA was isolated from dermal fibroblasts and analyzed via RT-qPCR. The expression of important developmental transcriptional regulators and signaling molecules implicated in fibroblasts’ growth, proliferation, and ECM production were quantified. To some extent, the mRNA expression of cell cycle progression regulators quantified with RT-qPCR corresponded to the protein expression data seen via Western blot. Cell proliferation inhibitor *CDKN2A*, encoding p16 was downregulated in aged CCD-1064Sk (*p* < 0.01, Figure 9A), but not in BJ-5ta fibroblasts (Figure S25). Although, *TP53* and p53 appeared to have unchanged expression in BJ-5ta senescent cells (*p* > 0.05, Figure S25 and Figure 7F respectively) and elevated in CCD-1064Sk (*p* > 0.05, Figure S26), it was not statistically significant. In contrast, protein p21, which is coded by gene *CDKN1A*, was higher in both prematurely aged BJ-5ta, but was only significant in the 50 µM of H_2_O_2_ treatment (*p* > 0.05, Figure 7E) and mRNA expression of *CDKN1A* was not significantly higher in BJ-5ta (*p* > 0.05, Figure S25) and CCD-1064Sk cells (*p* > 0.05, Figure S26) compared to untreated ones.

The senescence-associated abatement in transcript levels of ECM genes *COL1A1* and *COL3A1* was detected (*p* < 0.01, Figure 8A and Figure 8B, respectively), while *ELN* expression had no apparent change to untreated cells (*p* > 0.05, Figure 8C), which corresponds to the Western blot findings (Figure 7C) in BJ-5ta prematurely senescent fibroblasts. The results for the main dermal scaffold genes *COL3A1* in the CCD-1064Sk fibroblast line matched the results for the BJ-5ta fibroblast line (Figure 9B), while *ELN* expression appears to have higher expression when treated with hydrogen peroxide (Figure 9C). Interestingly, the *MMP1* (matrix metalloproteinase 1) gene, whose protein is known to degrade collagens and other components of ECM, was downregulated in aged cells (*p* < 0.05, Figure 8D). Similar alterations in gene expression were detected in prematurely aged CCD-1064Sk fibroblasts for *MMP2* (*p* < 0.001, Figure 9D). Another important gene associated with the maintenance of ECM and known to preserve components of the extracellular matrix from damaging activity of MMPs is *TIMP1* (tissue inhibitor of metalloproteinases 1). In addition, the encoded protein can promote cell proliferation by exhibiting an anti-apoptotic function. As expected, *TIMP1* was downregulated in CCD-1064Sk senescent fibroblasts (*p* < 0.01, Figure 9E), and decreased *TIMP1* would cause increased MMP functional activity resulting in decreased collagen production. Furthermore, *HAS1* (hyaluronan synthase 1 or hyaluronic acid), a vital component of ECM that provides skin hydration and supports the ECM, enabling fibroblasts to migrate through it [52], showed an unexpected rise in senescent cells (*p* > 0.05, Figure S26) but this change was not statistically significant.

Taken together, there was a change in the expression of numerous genes when comparing young to old passages of untreated cells and prematurely aged fibroblasts. Specifically, altered expression of p16, p21, and collagens were demonstrated and are indicators of SIPS. Furthermore, protein changes depended on both the model used and the fibroblast cell line.

### 2.6. Senescence-Related Changes in the Expression of Genes and Proteins Involved in Cell Cycle Regulation, Cellular Replication, and Metabolic Responses

To obtain an understanding of what molecular pathways were involved in the progression of senescence or may be abnormally regulated during senescence, we analyzed mRNA expression by RT-qPCR and protein levels by Western blot in dermal fibroblasts.

**Figure 10:**
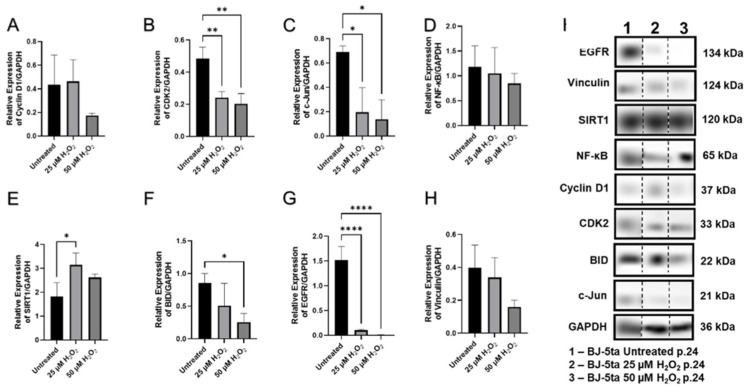
The expression of cell-cycle regulators, functional, and regulatory proteins in aged fibroblasts of BJ-5ta (p.24) cell line. Fibroblasts were aged by the one-step H_2_O_2_ senescence model. Figures represents changed protein expression levels for selected genes measured by Western blot. (**A**) Cyclin D1 expression; (**B**) CDK2 expression; (**C**) c-Jun expression; (**D**) NF-κB expression; (**E**) SIRT1 expression; (**F**) BID expression; (**G**) EGFR expression; (**H**) Vinculin expression. Glyceraldehyde-3-phosphate dehydrogenase (GAPDH) was used as a loading control. Relative densitometry was presented as a ratio of target protein to GAPDH (**I**). Data were analyzed with a one-way ANOVA test (Tukey post-hoc multiple comparison test). Significance (p) is indicated within the figures using the following scale: *, *p* < 0.05; **, *p* < 0.01; ****, *p* < 0.0001. Bars represent mean ± SD. The samples for each protein were run on the same gel (Figures S17–S24). Since the samples were not run consecutively on the same gel, they were spliced for presentation purposes only. No image enhancements were applied.

The expression of each cell was used in the experimental unit. Nuclear factor-kappa B showed a decreasing trend in both BJ-5ta and CCD-1064Sk prematurely aged fibroblasts (*p* > 0.05, Figure S25 and Figure S26, respectively). Similar trends were seen in Western blot analysis (Figure 10D). NF-κB is a protein transcription factor that controls transcription of DNA, cytokine production, and regulation of expression of multiple genes associated with cell survival, proliferation, and differentiation. Due to direct regulation on downstream gene expression, altered NF-κB protein expression would cause dysregulation of expression for numerous genes. Another critical gene, *EGFR* (epidermal growth factor receptor), was mildly upregulated (*p* > 0.05, Figure S25) and was poorly expressed on the protein level (*p* < 0.001, Figure 10G) after the H_2_O_2_ exposure, which is involved in cell signaling pathways that control cell division and survival.

Besides their role in cell signaling pathways that control cell division and survival, NF-κB and EGFR are also involved in apoptosis pathways, hence, deviations in their regulation might influence the process of programmed cell death and potentially senescence. For this reason, we tested the expression levels of the BH3 interacting-domain death agonist (BID), which is a pro-apoptotic member of the Bcl-2 protein family contributing to the mitochondrial pathway of apoptosis. Of note, BID expression demonstrated a dose-dependent decrease with exposure to increasing concentrations of H_2_O_2_ (Figure 10F). Corresponding changes were found in the level of vinculin expression (Figure 10H). Vinculin is a membrane-cytoskeletal protein associated with maintaining cell–cell and cell–matrix adhesion, emerging as a regulator of apoptosis, and found to be overexpressed in apoptotic cells [53]. c-Jun, which is part of the activator protein-1 (AP-1) complex involved in regulation of proliferation, apoptosis, survival, tumorigenesis, and tissue morphogenesis [54], was significantly diminished in aged fibroblasts (Figure 10C). Moreover, c-Jun is required for progression through the G1 phase of the cell cycle which occurs by a mechanism that incorporates direct transcriptional control of the cyclin D1 gene, forming a molecular connection between growth factor signaling and cell cycle regulation [55]. Cyclin D1 protein levels were almost identical to the untreated fibroblasts after treatment with 25 µM of H_2_O_2_ and insignificantly declined after treatment with 50 µM of H_2_O_2_ (Figure 10A). Importantly, the levels of cyclin-dependent kinase 2 (CDK2), a protein kinase which is activated by cyclin binding and enables transition from G1 to S phase, were significantly decreased (*p* < 0.05, Figure 10B) in both H_2_O_2_ treatment groups compared to the untreated aged BJ-5ta fibroblasts.

Changes in the cell cycle progression typically affect other cellular functions such as proliferation, differentiation, and metabolic activity. Recently ascertained growth differentiation factor 11 (GDF11), a key to progenitor proliferation and/or differentiation, was also considered to be important for the preservation of youthful phenotypes in different human tissues, and to inhibit inflammatory responses in the skin [56]. The expression of *GDF11* in prematurely aged fibroblasts tends to increase compared to untreated CCD-1064Sk fibroblast cells (*p* > 0.05, Figure S26). Another essential nuclear protein associated with cellular proliferation that is a marker of proliferation is Ki-67 (MKI67). *MKI67* was insignificantly elevated in fibroblasts treated with H_2_O_2_ (Figure S26).

Sirtuins (SIRT1–7) are known to prevent diseases and even reverse some aspects of aging. They are regulated at the level of transcription, translation, protein stability, and oxidation [57]. Notably, sirtuins can be grouped by location: nuclear sirtuins (SIRT1, 6, 7), cytosolic sirtuins (SIRT2), and mitochondrial sirtuins (SIRT3-5) [58,59,60,61]. Sirtuins have a multitude of functions including modulating energy metabolism, cell survival, cell cycling, DNA repair, tissue regeneration, inflammation, neuronal signaling, autophagy, mitochondrial biogenesis, oxidative stress, and acting as transcription factors [62,63,64,65,66,67].

The results of RT-qPCR analysis of sirtuins showed that *SIRT1* was mildly upregulated in aged CCD-1064Sk cells (*p* > 0.05, Figure S26), while in BJ-5ta fibroblasts it was downregulated (*p* > 0.05, Figure 8E). However, levels of protein expression of SIRT1 were significantly augmented in BJ-5ta fibroblasts exposed to the 25 µM H_2_O_2_ (Figure 10E). At the same time, *SIRT3* mRNA expression was strongly downregulated (*p* < 0.05, Figure 9F) in contrast to moderately upregulated *SIRT4* and *SIRT6* (*p* > 0.05, Figure 9G,H, respectively) in CCD-1064Sk. In BJ-5ta fibroblasts, only *SIRT4* mRNA expression was downregulated in the 25 µM of H_2_O_2_ treatment (Figure 8F). Interestingly, the mitochondrial SIRTs appear to be most affected, potentially due to increased mitochondrial ROS induced by H_2_O_2_ treatment.

In summary, changes in gene expression were observed when comparing young and senescent dermal fibroblasts. Along with the altered expression of numerous cell cycle regulators and genes with previously determined age-related expression changes, we found changes in the expression of proteins involved in metabolic regulation and the ECM.

### 2.7. Aspects of Senescence-Associated Cellular Viability

**Figure 11:**
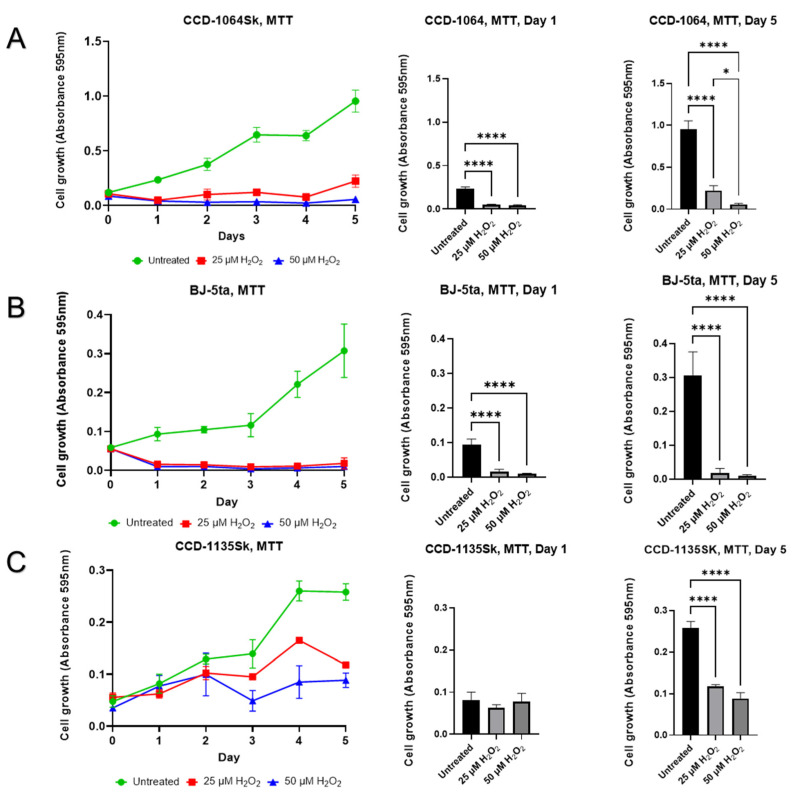
Viability of dermal fibroblasts estimated by MTT assay five days after a single treatment of 25 µM and 50 µM of H_2_O_2_ (one-step senescence model). (**A**) CCD-1064Sk, p.11; (**B**) BJ-5ta, p.42; (**C**) CCD-1135Sk, p.12. Data were analyzed with a one-way ANOVA test (Tukey post-hoc multiple comparison test). Significance (p) is indicated within the figures using the following scale: *, *p* < 0.05; ****, *p* < 0.0001. Bars represent mean ± SD.

Results of the cellular viability assay showed a significant reduction in the number of prematurely senescent fibroblasts (Figure 11). Cell viability of CCD-1064Sk cells were four times that of untreated newborn fibroblasts. At the same time, in the one-step model, cellular viability increased 20.8% after five days in the H_2_O_2_-induced senescent cells (Figure 11A).

Significantly less viability was detected in the prematurely senescent cells by approximately ten-fold compared to the untreated fibroblasts of the BJ-5ta cell line (Figure 11B). The development of senescent BJ-5ta fibroblasts stopped. Only a 3% increase in cell number was noticed five days after the H_2_O_2_ exposure.

In the adult dermal fibroblasts (CCD-1135Sk, p.12), we found decreased cellular viability (Figure 11C). Interestingly, the viability of untreated CCD-1135Sk was substantially lower than other cell lines, potentially since adult fibroblasts proliferate less than neonatal fibroblasts. At day five, the untreated skin CCD-1135Sk fibroblasts have significantly increased cellular viability compared to the prematurely H_2_O_2_-induced senescent cells (Figure 11C). At the same time, in CCD-1135Sk fibroblast, 25 µM H_2_O_2_ caused 2-fold decline in the viability, while 50 µM—approximately three-fold, as compared to untreated control. This interesting finding showed that adult CCD-1135Sk dermal fibroblasts at passage 12, which according to the manufacturer, have already completed around 30–34 population doublings (PD), are almost in a pre-senescent state as they show some signs of senescence [46,47].

### 2.8. Senescent Fibroblasts Showed Reduced Ability in the Healing Process

To determine if senescent dermal fibroblasts have a similar ability to fully participate in the healing process, we performed the wound healing assay in the adherent cellular monolayer of CCD-1064Sk and BJ-5ta cells. Images of the wound healing assay were taken at the following time points: 1 h, 6 h, 24 h, 48 h, and 72 h after scratching.

**Figure 12:**
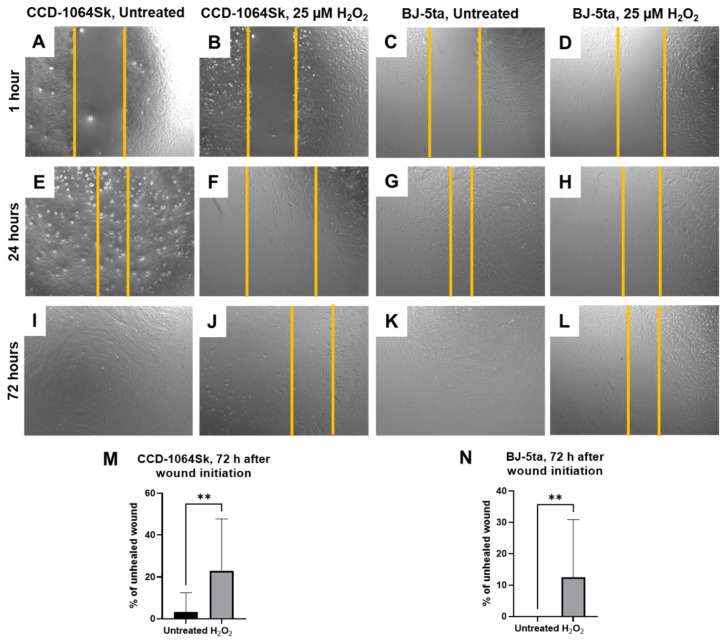
Regenerative ability of dermal fibroblasts estimated by wound-healing assay (one-step senescence model). Wound area in CCD-1064Sk, p.11 (**A**,**B**) and BJ-5ta, p.42 (**C**,**D**) one hour after single-time 25 µM H_2_O_2_ exposure. Decreased wound area in CCD-1064Sk, p.11 (**E**,**F**) and BJ-5ta, p.42 (**G**,**H**) 24 h after a single treatment of 25 µM of H_2_O_2_. Wound closure in CCD-1064Sk, p.11 (**I**,**J**) and BJ-5ta, p.42 (**K**,**L**) 72 h after a single treatment of 25 µM of H_2_O_2_. The percentage of the area of the wound that is not healed in CCD-1064Sk, p.11 (**M**) and BJ-5ta, p.42 (**N**) 72 h after single-time of 25 µM H_2_O_2_ exposure. Data were analyzed with a Student’s unpaired t-test for each separate parameter. **, *p* < 0.01. Bars represent mean ± SD.

The most prominent findings were detected at 24 h and 72 h after the beginning of the experiment. Thus, in both untreated cell lines, the wound was more than 50% closed after 24 h (Figure 12E,G). In contrast, the wound surface in the H_2_O_2_-exposed cell cultures was moderately increased (Figure 12F) or slightly narrowed (Figure 12H) at the same time point. Complete closure of the scratch line was observed 48 h after the beginning of the experiment in the BJ-5ta cell line (data not shown). Whereas after 72 h, about 3% of the wound area in the untreated CCD-1164Sk fibroblasts and 23% (*p* < 0.01) in prematurely senescent fibroblasts were still uncovered (Figure 12M). In the case of BJ-5ta cells treated with H_2_O_2_, after 72 h, 12.5% of the wound area remained unhealed (*p* < 0.01, Figure 12N).

The wound-healing assay results demonstrated delayed regenerative capabilities of the senescent dermal fibroblasts, resulting in a decline in the healing process.

## 3. Discussion

Senescence has been studied for many decades, and still, there are more questions than answers. The main breakthroughs in cellular senescence were made after the Hayflick limit discovery. To date, we have accumulated knowledge about cell-cycle life span, gene function, regulation, editing, and molecular mechanisms of various pathways for multiple physiological and pathological processes, i.e., immune response, inflammation, senescence, and cancer regulation. However, we cannot stop senescence and have not even come close to understanding mechanisms that might allow us to delay it.

In our research, we focused on establishing a reliable model of cellular senescence. It is a well-known fact that the number of senescent cells increases with age. Considering that skin is the largest organ in the body and represents the main line of defense, it also expresses the first visible aging features. Accordingly, the most suitable cells for senescence studies in vitro are dermal fibroblasts, dominant in the skin structure and important for maintaining the skin’s microenvironment.

The three different fibroblasts cell lines used herein were selected for senescence model reproduction induced by H_2_O_2_ exposure. After H_2_O_2_ exposure, dermal fibroblasts exhibited senescent-like morphology, significantly higher β-Gal expression, decreased PD levels, and proliferation (Figure 3, Figure 4, Figure 5 and Figure 6). These findings are in line with previous reports, which show that SIPS is associated with growth arrest, elevation in the proportion of β-Gal-positive cells, and typical structural changes [19,22,36,42,68,69,70,71]. It is important to note that β-Gal is a lysosomal enzyme that is typically active at pH 4, but in RS and SIPS cells becomes detectable at pH 6 mirroring to some extent the increase in lysosomal mass [19]. It has been confirmed that lysosomes increase in number and size in senescent cells [72]. In 1995, Debacq-Chainiaux and colleagues found that an increase in β-galactosidase activity was strongly associated with senescent cells, as a function of replicative age both in the absence and presence of lysosomal inhibitors; β-Gal activity was not detected in quiescent or terminally differentiated cells [50]. Later on, Lee et al. confirmed the lysosomal origin of β-Gal activity when showed that it resulted from an increased expression of *GLB1*, the gene encoding lysosomal β-D-galactosidase, the activity of which is typically measured at acidic pH 4.5 [51].

Images taken 24 h after the beginning of exposure of H_2_O_2_ (Figure S3C,F,I,L) indicated a dose-dependent decline in the number of viable lysosomes containing NR dye. It might be related to the elevated degradation processes inside the prematurely aged fibroblasts and consequent reduction in the total number of viable cells. This would be in line with the MTT results, which showed a significant decrease in viability, suggesting a decrease in the rate of fibroblasts growth (Figure 11), which is supported by data from other research groups [3,73,74,75].

Apart from reduced viability, morphological changes in senescent dermal fibroblasts were also detected. Increased cellular size and changes in the cell shape were observed parallel to the changes in nucleus. The analysis of the prematurely aged fibroblast nuclei reflected morphological alterations of an entire cell. Nuclei of senescent fibroblasts displayed wide-scale deformations and lobulations, whereas young cells had smooth disk-shaped nuclei. Interestingly, nuclei from aged cells showed an increased area in both cell lines and significant busting of circularity that represents roundness (Figure 5 and Figure 6). At the same time, the gene expression and protein levels of ECM components, mainly collagens, which externally affect the cellular shape [76], were decreased.

Furthermore, such deviations in cellular shape might be caused by decreased expression of membrane-cytoskeletal protein vinculin in senescent cells, which plays a role in cell–cell and cell–matrix connections (Figure 10H). We can speculate that fibroblast enlargement was a compensatory response to a decline in vinculin level to prevent cellular detachment and increase the surface of adhesion. Other publications partially support these assumptions. Aifuwa and colleagues recently determined that prematurely senescent cells were considerably softer and less elastic than control cells [77]. The authors emphasized diminished focal adhesion (vinculin) and decreased expression of cytoskeletal proteins (lamin A/C, F-actin, myosin II, Rho A), as well as a significant reduction in root mean square traction forces and the cytoskeletal contractility of senescent cells that eventually result in the loss of cellular tension that correlated with shape changes of the cells and their nuclei. Altogether, these results may suggest that intercellular contractility and contact inhibition are sufficient to recapitulate the nuclear phenotype induced by H_2_O_2_ and potentiated by alterations in cell-cycle regulation followed by metabolic changes.

We observed increased transcriptional abundance from p21, deviations in p16 and p53, and decreased CDK2 and c-Jun, suggesting that the H_2_O_2_-dependent SIPS contribute to growth arrest typical for senescent cells (Table 2). Indeed, SIPS-triggered senescence is directly maintained by p53, which promotes the cyclin-dependent kinase inhibitor p21 upregulation, which in turn inhibits the action of CDK2 kinase activity arresting the cell cycle in G1. Additionally, p21 blocks the cyclin D1/CDK4/6-mediated hyperphosphorylation of Rb protein. The hypophosphorylated state of Rb leads to the inhibition of the transcription factor gene *E2F* and subsequent transcription machinery required for S-phase onset [19]. Moreover, p16 may mediate the initiation of H_2_O_2_-induced cell cycle arrest by inhibiting the activation of CDK4 and CDK6 that contribute to cell cycle phase progression ensuing repression of E2F target genes forcing a G1 cell-cycle arrest [73].

Our results indicated a significant reduction in CDK2, c-Jun protein levels, accompanied by a decline in cyclin D1 (Figure 11A,B), suggesting potential growth arrest. Interestingly, Morris and coworkers found that reduced cyclin D1 levels and enhanced association of p27kip1 with CDK2 causes G1 arrest in NIH 3T3 cells [78]. Another research group showed that in contrast to young cells, senescent fibroblasts contained mainly unphosphorylated cyclin E and proportionally more unphosphorylated and inactive CDK2. Moreover, in most senescent cells, the CDK2 was complexed with cyclin D1. It is worth mentioning that cyclin D1-CDK2 complexes were severalfold higher in senescent cells and contained exclusively unphosphorylated CDK2, perhaps accounting for the low kinase activity [79].

A critical step in the cell-cycle regulation is c-Jun-mediated G1 cell cycle progression, directly controlling cyclin D1 gene transcription [55]. Our experimental model showed a substantial reduction of c-Jun expression in SISP-induced fibroblasts (Figure 10C), which also explains the consequent alterations in CDK2 and cyclin D1 expression. Apart from the fact that c-Jun is involved in multiple molecular pathways, it also protects cells from various SIPS-associated stressors and their effects (i.e., UV-induced cell death) and cooperates with NF-κB to prevent apoptosis induced by tumor necrosis factor alpha (TNFα) [55]. At the same time, our data indicated that expression of *NF-κB* has been moderately downregulated in prematurely aged dermal fibroblasts (Figure S26). This might be an additional factor leading to delayed cell cycle progression as NF-κB, apart from controlling multiple genes associated with cell survival, proliferation, apoptosis, and differentiation, also activates the expression of cyclin D1.

Aside from the findings related to the altered cell-cycle regulators in SIPS-induced cells, changes in the proteins associated with apoptosis pathways (BID), metabolic regulation (sirtuins), and components of ECM (collagens, elastin, hyaluronan) were also found (Table 2). These results are widely supported by similar findings in various cell types and tissues [52,75,80,81,82,83,84,85,86].

Dermal fibroblasts are mesenchymal cells considered to maintain skin integrity and functionality via ECM production. They also orchestrate tissue repair by interacting with and controlling other cell types, including immune cells, myocytes, and keratinocytes in the wound microenvironment. Decreased expression of *COL1A1*, *COL3A1*, *TIMP1*, and elevation of elastin suggests degradation of ECM and explains delayed healing process and corresponds to the literature [82,85]. Moreover, collagen fibrils and elastin are responsible for the strength and resiliency of skin. It is a known fact that ECM component degeneration with aging causes the skin to become fragile and easily bruised. On the histological level, connective tissue damage (e.g., skin) induced by ultraviolet irradiation manifests as a disorganization of collagen fibrils resembling wavy worm-like appearance of the fibers, massive aggregation of abnormal, amorphous, and elastin containing material. Such accumulation of elastotic material (i.e., increased deposition of degraded elastin material) is accompanied by degeneration of the surrounding collagen matrix [87]. In support of the previous statements, we observed increased expression of elastin (Figure 9 and Figure 10) accompanied by a delayed wound healing process in the monolayer of cultured fibroblasts exposed to H_2_O_2_ (Figure 12). This data showed significant deterioration in regenerative capabilities of the prematurely aged fibroblasts that reflected the morphological, biomolecular, and functional changes in naturally old cells [19,88].

## 4. Conclusions

To summarize, both H_2_O_2_-induced senescence models designed in our lab clearly demonstrated visible changes in fibroblast morphology typical for SIPS corresponding to other research groups [19,68,89,90]. Moreover, both SIPS models showed the evidence of fibroblasts developing similar aging characteristics as the RS cells, compared to the respective young and untreated controls. Among the most noticeable senescent biomarkers were the increased β-Gal activity, accompanied by high p21 protein levels, altered levels of cell-cycle regulators (i.e., CDK2 and c-Jun) and ECM components, reduced cellular viability, and delayed wound healing properties (Table 2). This suggests that although replicative and H_2_O_2_-induced senescence present very similar phenotypic traits, pathogenetic factors in the SIPS model led to more apparent transcriptional changes.

Although all three cell lines are affected by hydrogen peroxide administration as noted by cell viability, the CCD-1135Sk adult dermal fibroblast cell line appeared to be least affected by hydrogen peroxide administration, with no apparent change after day one. Since this cell line is derived from adult fibroblasts instead of neonatal or immortalized fibroblasts, CCD-1135Sk may already display a quasi-senescent phenotype which masks the effects of hydrogen peroxide administration on senescent markers. Further study of CCD-1064Sk and BJ-5ta showed H_2_O_2_ administration caused increased nuclear area, perimeter, and min/max caliper lengths in CCD-1064Sk fibroblasts, whereas H_2_O_2_ administration caused increased circularity and decreased eccentricity in BJ-5ta fibroblasts. As BJ-5ta fibroblasts are immortalized via hTERT to prevent telomere degradation, it is not surprising there are differences in H_2_O_2_-induced changes of nuclei architecture. Although similar proteins and RNAs were affected as shown by Western blot and RT-qPCR analysis, different members of these families were affected. For example, H_2_O_2_ administration decreased COL1A1 protein expression and decreased *MMP2* and *SIRT3* RNA expression in CCD-1064Sk fibroblasts, whereas H_2_O_2_ administration decreased COL3A1 protein expression and *MMP1* and *SIRT4* RNA expression in BJ-5ta fibroblasts. Regardless, both cell lines demonstrated typical phenotypes of senescence and make reliable models of SIPS.

The results of our research uncovered potential mechanisms of cellular senescence, important differences among different fibroblast cell lines, and might help find possible anti-aging remedies. The one-step cellular senescence model developed here should be suitable to test effects of potential anti-aging compounds.

## 5. Materials and Methods

### 5.1. Cell Culture and Maintenance

Healthy human neonatal foreskin fibroblasts CCD-1064Sk (ATCC^®^ CRL-2076™), human adult skin fibroblasts CCD-1135Sk (ATCC^®^ CRL-2691™), and human foreskin BJ-5ta hTERT-immortalized cell lines (CRL-4001™) were obtained from the American Type Culture Collection (Rockville, MD, USA). Cells were cultivated in ISCOVE’s Modified Dulbecco’s Medium (IMDM) 1X (MULTICELL, Cat# 319-106-CL) containing 10% heat-inactivated Premium Grade Fetal Bovine Serum (Cat# 97068-085, VWR International LLC, Radnor, USA), and 1% Penicillin-Streptomycin (10,000 IU Penicillin and 10,000 µg/mL Streptomycin, Cat# 450-201-EL, WISENT INC., Saint-Jean-Baptiste, QC, Canada). All cells were grown and harvested in our BSL 2 laboratory at the University of Lethbridge. Experimental cell lines were incubated in a humidified Forma Steri-Cycle CO_2_ Incubator (Thermo Fisher Scientific, Waltham, MA, USA) at 37 °C with 5% CO_2_. Cell culture media were replaced with fresh media every three days until cell confluency reached 90–100% for further experiments. The cells were subcultured every six or seven days. The replication speed or population doubling (PD) numbers of the cell lines were determined for each subculture as ΔPD = log_2_(n_f_/n_i_), where n_i_ is the number of cells initially seeded and n_f_ is the final number of cells in a culture. Cells for the senescence model were not older than 24–30 population doublings when employed in the experiments.

### 5.2. Senescence-Associated Phenotype Modelling

#### 5.2.1. Three-Step Model of Skin Fibroblast Senescence

Newborn skin fibroblasts CCD-1064Sk were subcultured to passage 11 and exposed to H_2_O_2_ utilizing the three-step model (Figure 1). H_2_O_2_ was dissolved in the IMDM cell culture medium with 10% FBS in the following concentrations: 25 µM, 50 µM, 100 µM, 150 µM, 200 µM, 250 µM, 300 µM, and 350 µM. Each step started when cell confluency was approximately 70% with daily one-hour H_2_O_2_ exposure followed by complete fresh medium replacement. After 5 days of H_2_O_2_ treatment, cells were harvested and subcultured for the next step.

#### 5.2.2. One-step Hydrogen Peroxide Skin Fibroblast Senescence Model

Skin fibroblasts (CCD-1064Sk) at 70% confluency were treated for 1 h with 25 µM concentration of hydrogen peroxide solution (H_2_O_2_ dissolved in D-PBS) in 100 × 15 mm Petri plates in aseptic conditions (Figure 2). Petri plates with skin fibroblasts were maintained in a humidified incubator at 37 °C with 5% CO_2._

After a single 1-h treatment, H_2_O_2_ solution was poured out and substituted with cell culture medium. Subsequently, SIPS features and biomarkers were determined via microscopy, β-galactosidase senescence assay, MTT, Western immunoblotting, reverse transcription-polymerase chain reaction, wound-healing assay (WHA), and nuclear DAPI staining.

### 5.3. β-Galactosidase Analysis

The activity of β-galactosidase (β-Gal) in fibroblasts was detected by the beta-Galactosidase Detection Kit (Fluorometric) (ab176721, Abcam) following the manufacturer instructions. Briefly, cell lysate was prepared from normal and premature aged cells with H_2_O_2_ with protein lysis buffer (included in the kit). Sample protein concentration was quantified using a Bradford protein assay and samples were diluted to 1 µg/mL protein concentration. Then, 50 μL of the standard and samples (diluted in 1× lysis buffer) were added to 96-well black plates followed by 50 μL of fluorogenic fluorescein digalactoside (FDG) working solution to each well and incubated at 37 °C for 4 hours. After adding 50 μL of the stop buffer, fluorescence in each sample was quantified with a FLUOstar Omega (BMG LABTECH, Cary, NC, USA) filter-based multi-mode microplate reader at 490 nm for excitation and 525 nm for emission. Cell senescence was established by β-Gal levels in each sample utilizing a β-galactosidase standard curve prepared for each experiment. All experiments were repeated three times (*n* = 3); each test was done in triplicate.

### 5.4. Cell Viability/Cytotoxicity

#### 5.4.1. The Micro-Culture Tetrazolium Assay (MTT)

Cell viability of CCD-1064Sk, CCD-1135Sk, and BJ-5ta human skin fibroblasts was measured by the micro-culture tetrazolium (MTT, 3-[4,5-dimethylthiazol-2-yl]-2,5-diphenyltetrazolium bromide; thiazolyl blue) colorimetric metabolic activity assay with the cell proliferation kit I (#11465007001, Roche, ON, Canada) according to the manufacturer’s instructions.

Cells were plated at 3.0 × 10^3^ cells/well in 150 μL of cell culture medium in a 96-well assay plate and cultivated for 24–48 h before treatment depending on cell confluency. A broad range of H_2_O_2_ concentrations was examined to determine the appropriate effective/cytotoxic concentration for each designated treatment. Unless otherwise indicated, all measurements were performed in triplicate at specific time points (0, 1, 2, 3, 4, and 5 days). After the desired treatment time, 10 μL of MTT labeling reagent was added to each well without removing media and incubated for 4 h. Afterward, 100 μL of MTT solubilization solution (10% SDS in 0.01 M HCl) was added to each well, followed by overnight incubation. Cell viability was calculated by comparing it to the control treatment. All experiments were repeated three times (*n* = 3); each test was done in triplicate.

#### 5.4.2. Neutral Red Stain

The neutral red stain is based on the ability of viable cells to incorporate and bind neutral red dye in the lysosomes [91]. It was used to provide a qualitative estimation of the presence of viable cells in the fibroblast cell cultures.

Cells were cultivated in 24-well cell culture plates and treated appropriately. The medium was removed from the fibroblast cell cultures and the cultures were washed with PBS. After that, 100 μL of Neutral red (N7005, Sigma-Aldrich, Saint Louis, MO, USA) dissolved in a cell culture medium (40 μg mL^−1^) was added to each well, followed by 4 h incubation at the appropriate culture conditions. After incubation, cells were gently washed twice with 150 μL of PBS. Images were taken using a Zeiss Observer Z1 epifluorescence microscope with AxioVision Rel 4.8 software.

#### 5.4.3. Crystal Violet Stain

The viability of cultured fibroblasts was evaluated by detecting maintained adherence of cells by staining attached cells with crystal violet dye, which binds to proteins and DNA [92]. It is worth noting that a reduced amount of crystal violet staining in cell culture represents cells that undergo cell death and simultaneously lose their adherence and die.

Cells were cultivated in 24-well cell culture plates and treated appropriately. The medium was aspirated from the fibroblast cell cultures, and cells were washed twice with PBS. Then, 50 μL of 0.5% crystal violet staining solution was added to each well and incubated for 20 min at room temperature on a bench rocker with a frequency of 20 oscillations per minute. After incubation, plates were gently washed four times in a stream of tap water, and then were inverted on filter paper and tapped gently to remove any remaining liquid. Then, plates without their lids were left to air dry for at least 2 h at room temperature. Images were taken using a Zeiss Observer Z1 epifluorescence microscope with AxioVision Rel 4.8 software.

### 5.5. Protein Extraction and Quantification

The three cell lines of dermal fibroblasts were harvested by using TRYPSIN/EDTA (0.25% Trypsin and 2.21 mM EDTA-4Na, Cat#325-043-EL, WISENT INC., Saint-Jean-Baptiste, QC, Canada). The mixture was centrifuged at 1600 rpm for five min. The supernatant was discarded, and the pellets were washed twice with ice-cold 1× PBS. The pellet was solubilized in 100–150 μL RIPA lysis buffer with 10 mM Tris-HCl (pH 7.5), 100 mM NaCl, 1 mM EDTA, 1% Triton X-100, 10% glycerol, 0.1% SDS, 0.5% deoxycholate, 1 mM sodium orthovanadate, and 1 mM PMSF. Whole cellular protein lysate was sonicated using a Braunsonic model 1510 sonicator (B. Braun, Melsungen, Germany) operating at 80% sonication capacity. Lysates were centrifuged at 12,000× *g* for 10 min and the supernatant was decanted. Using the Bradford protein assay with bovine serum albumin as the standard, protein concentrations were determined via NanoDrop 2000/2000c Spectrophotometer (Thermo Fisher Scientific, Wilmington, DE, USA).

### 5.6. Western Immunoblotting

Western immunoblotting was conducted with 50 μg and was prepared with 4× loading buffer (0.0625 M Tris, 2% SDS, 10% glycerol, 0.01% bromophenol blue, and 1% 2-mercaptoethanol) and RIPA lysis buffer and heated at 95 °C for 10 min [93]. The protein sample and PageRuler Plus Prestained Protein Ladder (Cat#26620, Thermo Scientific, MA, USA) were loaded and electrophoretically separated by SDS-PAGE into slab gels of 10–15% polyacrylamide at 100 V. Polyvinylidene difluoride membranes (Amersham Biosciences, Baie d’Urfé, Québec) were used to transfer resolved proteins for 2 h on ice. Then, membranes were incubated for two hours in a blocking solution (5% dry skimmed milk in PBS, 0.5% Tween 20) at room temperature and incubated with specific primary antibodies specified in Table S1 at 4 °C overnight.

After overnight incubation, the membranes were washed three times with 0.1% Tween-20 in PBS (PBS-T). Then membranes were incubated with 1:10,000 dilution of either Bovine anti-mouse secondary antibody or Donkey anti-Rabbit secondary antibodies (Table S1) for two hours at room temperature.

Membranes were washed three times with PBS-T and then exposed to ECL Prime Western Blotting System (Cat#GERPN2232, GE Healthcare, Chicago, IL, USA). Chemiluminescence was detected using the FluorChem HD2 Imaging System (Cell Biosciences, Santa Clara, CA, USA). Unaltered PVDF membranes were stained with Coomassie blue (BioRad, Hercules, CA, USA) to confirm equal protein loading. Signals were quantified using the NIH Image J64 software and normalized relative to GAPDH or Coomassie staining as indicated.

### 5.7. RNA Isolation

RNA was isolated from monolayer fibroblast cultures, using TRIzol^®^ Reagent (Invitrogen, Carlsbad, CA, USA); purified using an RNAesy kit (Qiagen), according to the manufacturer’s instructions; and quantified using NanoDrop 2000c (Thermo Fisher Scientific, Wilmington, DE, USA).

### 5.8. Quantitative Real-Time PCR (RT-qPCR)

Quantitative real-time PCR (RT-qPCR) was performed on skin fibroblast samples from all experimental groups. According to the manufacturer’s instructions, cDNA was generated with 500 ng RNA using the iScriptTM Select cDNA synthesis kit (Cat# 1708897, BioRad, Hercules, CA, USA). PCR reactions were based on the SsoFastTM EvaGreen^®^ Supermix (Cat# 1725202, BioRad, Hercules, CA, USA) and 500 nM of forward and reverse primers specific for target sequences of interest. Primers were designed using the https://www.idtdna.com/Primerquest platform (accessed on 1 February 2021) (Table S2). Primers were checked before on dilution series of normal fibroblasts cDNA. Reference genes (GAPDH, RPL13A, and UBC) were analyzed with the GeNorm method [94]. The reactions were analyzed on a C1000TM Thermo Cycler equipped with a CFX96 Touch™ Real-Time PCR Detection System (BioRad, Hercules, CA, USA). The PCR programs were run according to the SSoFastTM guidelines with annealing temperatures as specified for the specific primer pairs. Expression analysis was performed with the BioRad Software (CFX Manager) and was based on the ΔΔCt method with the reference genes that were stably expressed in the GeNorm analysis. Each experiment included three biological replicates for each group and two technical replicates per sample.

### 5.9. Wound-Healing Assay

Cells were cultivated to >90% confluence in 24-well plates. Ten microliter pipette tips were used to scrape a scratch/wound line through the middle of each well simulating a wound. Cells were washed twice in PBS before adding cell culture growth medium or designated treatments. Images of the healing process were taken on the following time points: 1 h, 6 h, 24 h, 48 h, and 72 h throughout the assay.

The Infinity3 camera was used to collect images within the linear dynamic range representing the range in which the relationship between signal intensity and the amount of material is likely to be linear. Images were analyzed with ImageJ (IJ 1.46r) software. At least seven measurements were counted per sample, and samples were designed in triplicates.

### 5.10. Immunocytochemistry

Cells were plated on glass coverslips for 48 h, treated in 6-well plates, and then fixed in 3% formaldehyde for 20 min at room temperature. Cells were quenched with 50 mM NH_4_Cl in PBS, permeabilized for 5 min in 0.2% Triton X-100, and blocked with 3% BSA for 30 min. After washing, nuclei were stained with 300 nM 4′,6-diamidino-2-phenylindole (DAPI) (Thermo Fisher Scientific, Waltham, MA, USA, Cat #D1306) in PBS for 15 min before mounting according to the manufacturer’s instructions. Images were taken using a Zeiss Observer Z1 epifluorescence microscope with AxioVision Rel 4.8 software. DAPI produces a blue fluorescence when bound to DNA with excitation at 360 nm and emission at 460 nm. Specimens were stored at 4 °C. Experiments were prepared in triplicates.

### 5.11. QuPath Analysis

QuPath 0.2 was used for quantitative analysis of cell number and stained area for the nuclei of the fibroblasts [95]. Images were taken using a Zeiss Observer Z1 epifluorescence microscope with AxioVision Rel 4.8 software and imported into QuPath. Nuclei were quantified using the cell detection function on the region of interest with parameters optimized to identify nuclei accurately and were confirmed by visual inspection. Any nuclear overlapping or partial nuclei captured erroneously identified as nuclei were deleted. Once nuclei were correctly identified, QuPath provided the following parameters:

Nuclei number—the number of distinct nuclei that were identified.

Area of the nucleus (μm^2^)—the number of pixels that enclosed within the nuclear perimeter.

Perimeter of the nucleus (μm)—the number of adjacent pixels in the boundary of the nucleus.

Nucleus circularity—area-to-perimeter ratio which demonstrates the roundness of the nuclear perimeter. This is calculated by multiplying the area by four pi and divided by the square of the convex perimeter. For circular nuclei, the ratio equals 1, whereas nuclei that depart from circularity have ratios less than one.

Nuclear max caliper (μm)—the distance between farthest parallel endpoints touching opposite sides of the nucleus.

Nuclear min caliper (μm)—the distance between closest parallel endpoints touching opposite sides of the nucleus.

Nuclear eccentricity, or ellipticity—the ratio of the min caliper to the max caliper. It shows how oval-shaped the nuclei are.

All data were automatically converted from pixels to the appropriate units and were extracted from QuPath 0.2 software to Microsoft^®^ Office Excel 365 files. Data were imported into the GraphPad Prism software 9.3.1 for statistical analysis (GraphPad Software, San Diego, CA, USA).

### 5.12. Statistical Analysis

The number of cell passages and biological repeats (n) for each experiment are indicated in the figure captions. Results are presented as mean of at least three samples per group with standard deviation (SD) of the mean or 95% confidence interval as indicated. Mean values ± SD and statistical analyses were calculated and plotted using GraphPad Prism 9 (GraphPad Software, San Diego, CA, USA) unless stated otherwise. Statistical analysis of data quantification was performed using a one-way ANOVA test (Tukey post-hoc multiple comparison test) and an unpaired Student’s *t*-test was used for analysis with two groups. Significance (p) was indicated within the figures using the following scale: *, *p* < 0.05; **, *p* < 0.01; ***, *p* < 0.001; ****, *p* < 0.0001.

## Figures and Tables

**Table 1:** In vitro H_2_O_2_ stress-induced senescence models.

Effective H_2_O_2_ Dose(s)	Lethal Dose	Time of Exposure	H_2_O_2_ Solvent	Type of Cells	Senescence Markers	Additional Findings in Senescent Cells	Reference
25 μM	>50 μM	N/A	PBS	FSE cells, and BJ fibroblasts	↑ SA-β-Gal	No differences with varying cell density	[39]
50 μM	1 mM	30 min, every 2 days within 8 days	PBS	Human MRC5 fibroblasts	↑ SA-β-Gal	↓ *c-fos*; H_2_O_2_-induced DNA damage inversely correlated with GPx	[40]
100 µM and 200 µM	N/A	2 h	Cell culture medium	IMR-90 lung fibroblasts	Senescence morphology, ↑ SA-β-Gal, ↑ p16, p21, & caveolin-1	↑ *Bcl-2* gene with H4K16Ac, and ↓ with H4K20Me3, contributing to its apoptosis-resistant phenotype	[38]
200 µM	>300 μM	2 h	Cell culture medium	F65 diploid foreskin fibroblasts	Senescence morphology, ↓ ODC; ↓ TK	↓ number of PD by 35.3 +/- 10.3%; Catalase & Deferoxamine protected cells from H_2_O_2_-induced replicative cessation	[41]
800 µM	N/A	0–72 h	Cell culture medium with 10% FBS and supplemented with 5-AzazC (10 μM) or ROS inducer menadione (10 μM)	normal human epidermal keratinocytes	↑ SA-β-Gal; ↑ p16; p21; p53 was not affected.	↓ expression of phosphorylated Rb and CDK4, resulting in arrest in G0/G1 phase; menadione ↑ the expression of mRNA and protein of p16INK4a, when antioxidant drug N-acetylcysteine ↓ it	[42]

BSA, bovine serum albumin; CDKs, Cyclin-dependent kinases; FBS, fetal bovine serum; FSE, Human primary foreskin fibroblasts; GPx, glutathione peroxidase; H4K16Ac, H4K16 acetylation; H4K20Me3, H4K20 trimethylation; N/A, not applicable; ODC, ornithine decarboxylase; PBS, phosphate-buffered saline; PD, population doublings; SA-β-Gal, senescence-associate -β-galactosidase; TK, thymidine kinase.

**Table 2:** Summary of the investigated characteristics of H_2_O_2_-induced prematurely aged skin fibroblasts compared to untreated fibroblasts.

Characteristic	H_2_O_2_-Induced SIPS Fibroblasts
Cell shape & size	Enlarged, flattened, irregular
Nuclear circularity	Increased
β-Gal level	High
BID	Decreased
CDK2	Low
c-Jun	Low
Collagens (type I, III)	Low
Cyclin D1	No change detected
EGFR	Decreased
Elastin	Increased
Hyaluronan	Moderately increased
MMP 1, 2	Low
MKI67	High
NF-kB	No change detected
P16	Moderate
P21	High
P53	No change detected
SIRT 1	No change detected
SIRT 3, 4	Low
SIRT 6	No change detected
TIMP 1	Low
Vinculin	Low
Cell viability	Decreased
Wound healing	Decreased

## Data Availability

Original blots can be obtained in supplementary figures.

## Supplementary Materials

The following supporting information can be downloaded at: https://www.mdpi.com/article/10.3390/ijms23137124/s1.
