# Supplementary material for: Modeling of the Senescence-Associated Phenotype in Human Skin Fibroblasts
Source: Int J Mol Sci. 2022 Jun 27;23(13):7124. doi: 10.3390/ijms23137124 (PMC9266450; doi:10.3390/ijms23137124)
Supplement: Supplementary file 1 [file ijms-23-07124-s001.zip › ijms-1775102-supplementary.pdf]

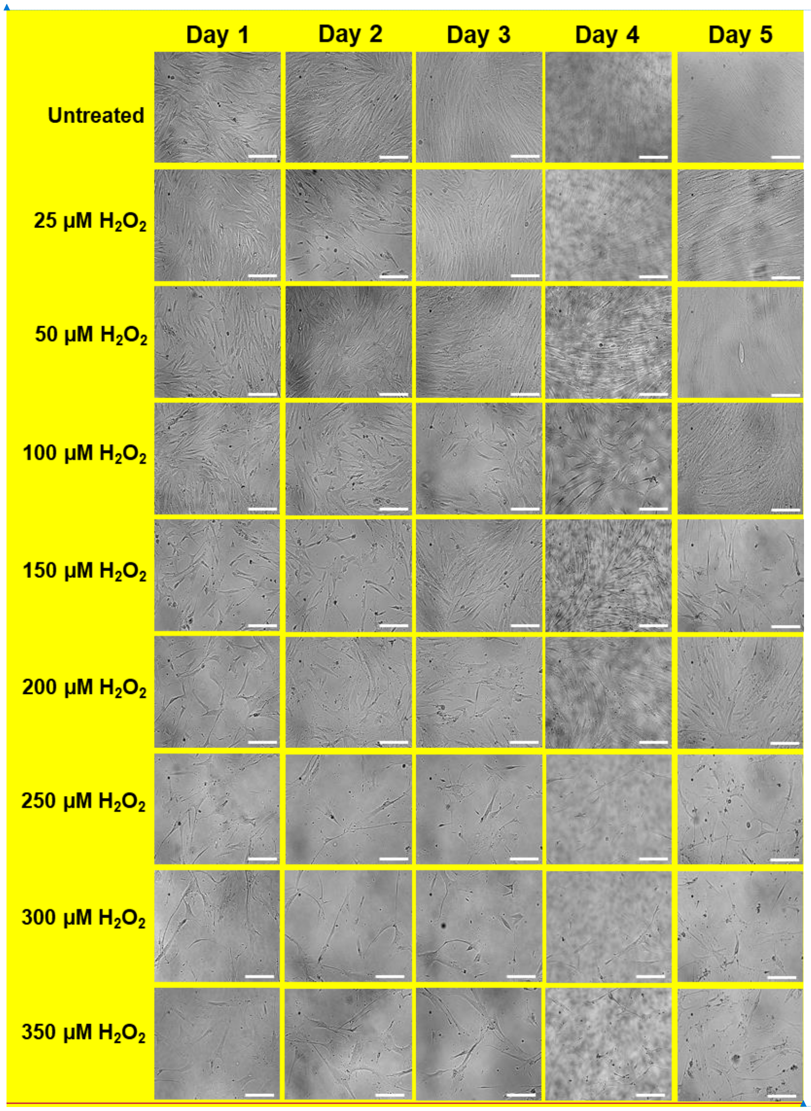

Figure S1. Human skin fibroblasts (CCD-1064Sk), p.11 exposed to H_2O_2 (Step 1 of the three-step senescence model). The figure represents gradual changes in cell quantity and quality within five days of H_2O_2 (doses of 25 µM, 50 µM, 100 µM, 150 µM, 200 µM, 250 µM, 300 µM, and 350 µM H_2O_2). Scale bars = 100 µm.

Formatted: Highlight

Formatted: Highlight

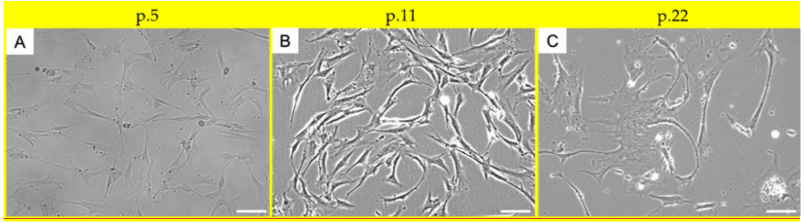

Figure S2. Human skin fibroblasts (CCD-1064Sk) at different passage numbers. A. "Early" passage of fibroblast cell culture (p.5); B. "Middle" passage of fibroblast cell culture (p.11); C. Replicative senescence (p.22). Scale bars = 100 µm.

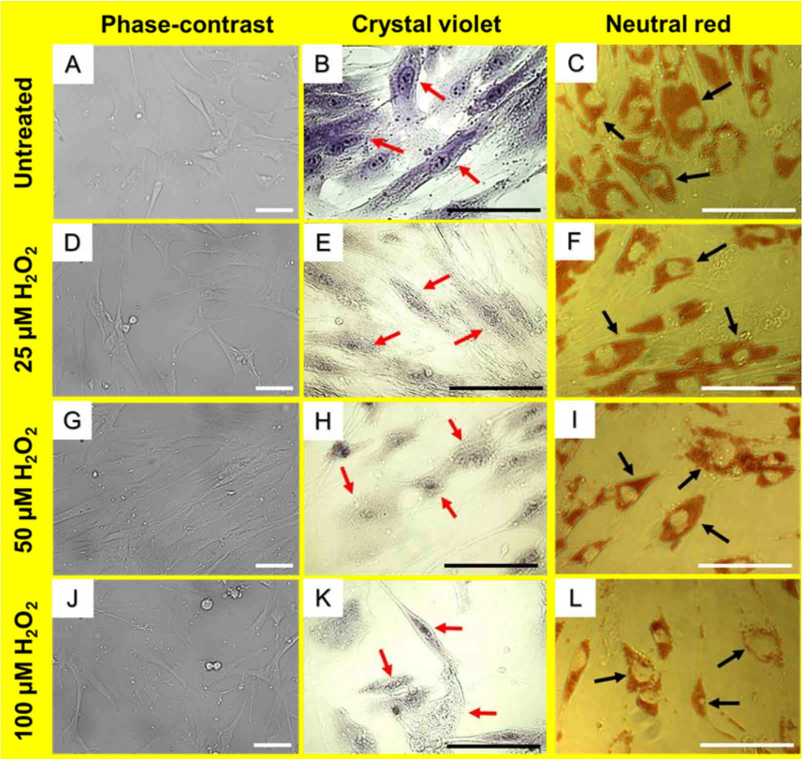

Figure S3. Human skin fibroblasts (CCD-1064Sk) p.11, 24 hours after 1-hour H_2O_2 exposure (One-step senescence model). Phase-contrast microscopy (A, D, G, J); Crystal violet staining (B, E, H, K); Neutral red staining (C, F, I, L), where Untreated (A-C); 25 µM H_2O_2 (D-F); 50 µM H_2O_2 (G-I); 100 µM H_2O_2 (J-L). Arrows point to specific staining accumulation: red – crystal violet, black – neutral red. Scale bars = 50 µm.

COL1A1 140-210 kDa

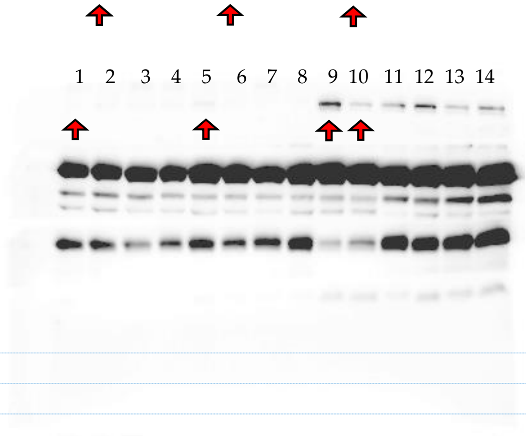

#	Sample
1	1135 p.18 Untreated
2	1135 p.18 DMSO
3	1135 p.18 THC 2.0 µM
4	1135 p.18 CBD 2.0 µM
5	1064 p.22 Untreated
6	1064 p.22 DMSO
7	1064 p.22 THC 2.0 µM
8	1064 p.22 CBD 2.0 µM
9	1064 p.11 Untreated
10	1064 p.11 H_2O_2 25 µM
11	1064 p.11 H_2O_2+DMSO
12	1064 p.11 H_2O_2+THC
13	1064 p.11 H_2O_2+CBD
14	1064 p.11 PBS

Figure S4. Western blot images of CCD-1135Sk p.18, CCD-1064Sk, p.22, and CCD-1064Sk, p.11. Original Western blots showing the COL1A1, Red arrows indicate bands shown in Figure 6.

Deleted: 1135 p18 Untreated
Deleted: 1135 p18 DMSO
Deleted: 1135 p18 THC 2.0 µM
Deleted: 1135 p18 CBD 2.0 µM
Deleted: 1064 p22 Untreated
Deleted: 1064 p22 DMSO
Deleted: 1064 p22 THC 2.0 µM
Deleted: 1064 p22 CBD 2.0 µM
Deleted: 1064 p11 Untreated
Deleted: 1064 p11 H_2O_2 25 µM
Deleted: 1064 p11 H_2O_2+DMSO
Deleted: 1064 p11 H_2O_2+THC
Deleted: 1064 p11 H_2O_2+CBD
Deleted: 1064 p11 PBS
Deleted: 1
Formatted: Highlight
Deleted: 7
Formatted: Highlight

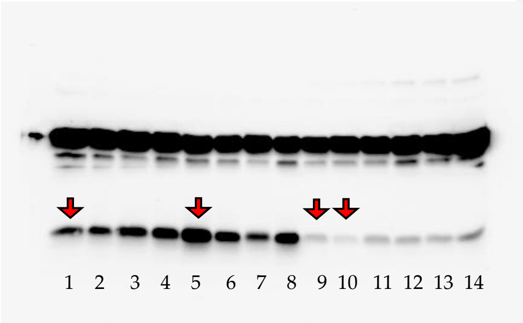

#	Sample
1	1135 p.18 Untreated
2	1135 p.18 DMSO
3	1135 p.18 THC 2.0 µM
4	1135 p.18 CBD 2.0 µM
5	1064 p.22 Untreated
6	1064 p.22 DMSO
7	1064 p.22 THC 2.0 µM
8	1064 p.22 CBD 2.0 µM
9	1064 p.11 Untreated
10	1064 p.11 H_2O_2 25 µM
11	1064 p.11 H_2O_2+DMSO
12	1064 p.11 H_2O_2+THC
13	1064 p.11 H_2O_2+CBD
14	1064 p.11 PBS

Figure S5. Western blot images of CCD-1135Sk p.18, CCD-1064Sk, p.22, and CCD-1064Sk, p.11. Original Western blots showing the p21 bands. Red arrows indicate bands shown in Figure 6.

Deleted: 1135 p18 Untreated
Deleted: 1135 p18 DMSO
Deleted: 1135 p18 THC 2.0 µM
Deleted: 1135 p18 CBD 2.0 µM
Deleted: 1064 p22 Untreated
Deleted: 1064 p22 DMSO
Deleted: 1064 p22 THC 2.0 µM
Deleted: 1064 p22 CBD 2.0 µM
Deleted: 1064 p11 Untreated
Deleted: 1064 p11 H_2O_2 25 µM
Deleted: 1064 p11 H_2O_2+DMSO
Deleted: 1064 p11 H_2O_2+THC
Deleted: 1064 p11 H_2O_2+CBD
Deleted: 1064 p11 PBS
Deleted: 2
Formatted: Highlight
Deleted: 7

p16 16 kDa

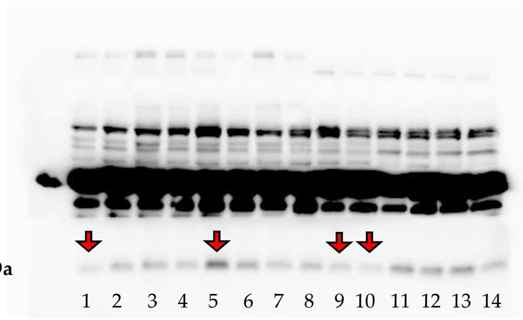

#	Sample
1	1135 p.18 Untreated
2	1135 p.18 DMSO
3	1135 p.18 THC 2.0 µM
4	1135 p.18 CBD 2.0 µM
5	1064 p.22 Untreated
6	1064 p.22 DMSO
7	1064 p.22 THC 2.0 µM
8	1064 p.22 CBD 2.0 µM
9	1064 p.11 Untreated
10	1064 p.11 H_2O_2 25 µM
11	1064 p.11 H_2O_2+DMSO
12	1064 p.11 H_2O_2+THC
13	1064 p.11 H_2O_2+CBD
14	1064 p.11 PBS

Figure S6. Western blot images of CCD-1135Sk p.18, CCD-1064Sk, p.22, and CCD-1064Sk, p.11. Original Western blots showing the p16 bands. Red arrows indicate bands used in Figure 6.

Deleted: 1135 p18 Untreated
Deleted: 1135 p18 DMSO
Deleted: 1135 p18 THC 2.0 µM
Deleted: 1135 p18 CBD 2.0 µM
Deleted: 1064 p22 Untreated
Deleted: 1064 p22 DMSO
Deleted: 1064 p22 THC 2.0 µM
Deleted: 1064 p22 CBD 2.0 µM
Deleted: 1064 p11 Untreated
Deleted: 1064 p11 H_2O_2 25 µM
Deleted: 1064 p11 H_2O_2+DMSO
Deleted: 1064 p11 H_2O_2+THC
Deleted: 1064 p11 H_2O_2+CBD
Deleted: 1064 p11 PBS
Deleted: 3
Formatted: Highlight
Deleted: 7
Formatted: Highlight

GAPDH 35 kDa

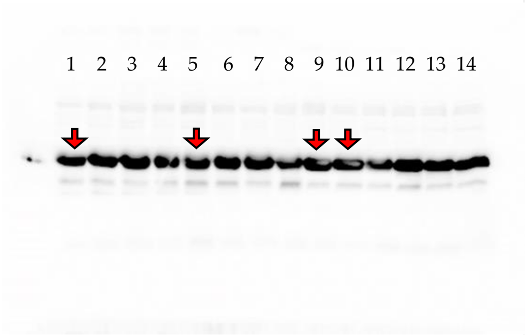

#	Sample
1	1135 p.18 Untreated
2	1135 p.18 DMSO
3	1135 p.18 THC 2.0 µM
4	1135 p.18 CBD 2.0 µM
5	1064 p.22 Untreated
6	1064 p.22 DMSO
7	1064 p.22 THC 2.0 µM
8	1064 p.22 CBD 2.0 µM
9	1064 p.11 Untreated
10	1064 p.11 H_2O_2 25 µM
11	1064 p.11 H_2O_2+DMSO
12	1064 p.11 H_2O_2+THC
13	1064 p.11 H_2O_2+CBD
14	1064 p.11 PBS

Figure S7. Western blot images of CCD-1135Sk p.18, CCD-1064Sk, p.22, and CCD-1064Sk, p.11. Original Western blots showing the GAPDH bands. Red arrows indicate bands used in Figure 6.

Deleted: 1135 p18 Untreated
Deleted: 1135 p18 DMSO
Deleted: 1135 p18 THC 2.0 µM
Deleted: 1135 p18 CBD 2.0 µM
Deleted: 1064 p22 Untreated
Deleted: 1064 p22 DMSO
Deleted: 1064 p22 THC 2.0 µM
Deleted: 1064 p22 CBD 2.0 µM
Deleted: 1064 p11 Untreated
Deleted: 1064 p11 H_2O_2 25 µM
Deleted: 1064 p11 H_2O_2+DMSO
Deleted: 1064 p11 H_2O_2+THC
Deleted: 1064 p11 H_2O_2+CBD
Deleted: 1064 p11 PBS
Deleted: 4
Formatted: Highlight
Deleted: 7
Formatted: Highlight

1 2 3 4 5 6 7 8 9 10 11 12 13 14

Elastin 70 kDa

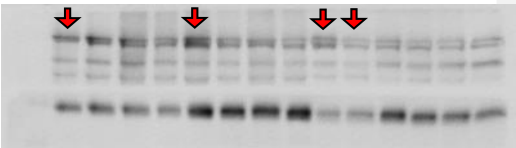

#	Sample
1	1135 p.18 Untreated
2	1135 p.18 DMSO
3	1135 p.18 THC 2.0 µM
4	1135 p.18 CBD 2.0 µM
5	1064 p.22 Untreated
6	1064 p.22 DMSO
7	1064 p.22 THC 2.0 µM
8	1064 p.22 CBD 2.0 µM
9	1064 p.11 Untreated
10	1064 p.11 H_2O_2 25 µM
11	1064 p.11 H_2O_2+DMSO
12	1064 p.11 H_2O_2+THC
13	1064 p.11 H_2O_2+CBD
14	1064 p.11 PBS

Figure S8. Western blot images of CCD-1135Sk p.18, CCD-1064Sk, p.22, and CCD-1064Sk, p.11. Original Western blots showing the Elastin bands. Red arrows indicate bands used in Figure 6.

Deleted: 1135 p18 Untreated
Deleted: 1135 p18 DMSO
Deleted: 1135 p18 THC 2.0 µM
Deleted: 1135 p18 CBD 2.0 µM
Deleted: 1064 p22 Untreated
Deleted: 1064 p22 DMSO
Deleted: 1064 p22 THC 2.0 µM
Deleted: 1064 p22 CBD 2.0 µM
Deleted: 1064 p11 Untreated
Deleted: 1064 p11 H_2O_2 25 µM
Deleted: 1064 p11 H_2O_2+DMSO
Deleted: 1064 p11 H_2O_2+THC
Deleted: 1064 p11 H_2O_2+CBD
Deleted: 1064 p11 PBS
Deleted: 5
Formatted: Highlight
Deleted: 7
Formatted: Highlight

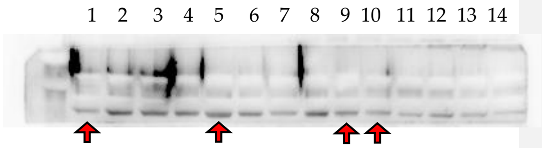

#	Sample
1	1135 p.18 Untreated
2	1135 p.18 DMSO
3	1135 p.18 THC 2.0 µM
4	1135 p.18 CBD 2.0 µM
5	1064 p.22 Untreated
6	1064 p.22 DMSO
7	1064 p.22 THC 2.0 µM
8	1064 p.22 CBD 2.0 µM
9	1064 p.11 Untreated
10	1064 p.11 H_2O_2 25 µM
11	1064 p.11 H_2O_2+DMSO
12	1064 p.11 H_2O_2+THC
13	1064 p.11 H_2O_2+CBD
14	1064 p.11 PBS

Figure S9. Western blot images of CCD-1135Sk p.18, CCD-1064Sk, p.22, and CCD-1064Sk, p.11. Original Western blots showing the p53 bands. Red arrows indicate bands used in Figure 6.

Deleted: 1135 p18 Untreated

Deleted: 1135 p18 DMSO

Deleted: 1135 p18 THC 2.0 µM

Deleted: 1135 p18 CBD 2.0 µM

Deleted: 1064 p22 Untreated

Deleted: 1064 p22 DMSO

Deleted: 1064 p22 THC 2.0 µM

Deleted: 1064 p22 CBD 2.0 µM

Deleted: 1064 p11 Untreated

Deleted: 1064 p11 H_2O_2 25 µM

Deleted: 1064 p11 H_2O_2+DMSO

Deleted: 1064 p11 H H_2O_2+THC

Deleted: 1064 p11 H_2O_2+CBD

Deleted: 1064 p11 PBS

Deleted: 6

Formatted: Highlight

Deleted: 7

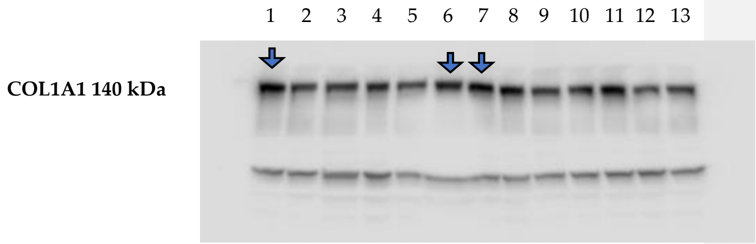

#	Sample
1	Untreated
2	PBS
3	DMSO
4	THC 2.0 μM
5	CBD 2.0 μM
6	H_2O_2 25 μM
7	H_2O_2 50 μM
8	H_2O_2 25 μM +DMSO
9	H_2O_2 50 μM +DMSO
10	H_2O_2 25 μM +THC
11	H_2O_2 50 μM +THC
12	H_2O_2 25 μM +CBD
13	H_2O_2 50 μM +CBD

Figure S10. Western blot images of BJ-5ta, p.24. Original Western blot showing the COL1A1 bands. Blue arrows indicate bands used in Figure 7.

Deleted: 7

Formatted: Check spelling and grammar, Highlight

Deleted: 8

Formatted: Highlight

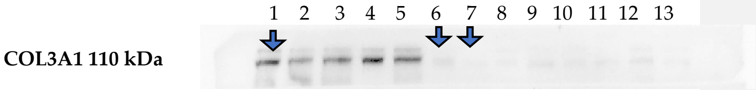

#	Sample
1	Untreated
2	PBS
3	DMSO
4	THC 2.0 μM
5	CBD 2.0 μM
6	H_2O_2 25 μM
7	H_2O_2 50 μM
8	H_2O_2 25 μM +DMSO
9	H_2O_2 50 μM +DMSO
10	H_2O_2 25 μM +THC
11	H_2O_2 50 μM +THC
12	H_2O_2 25 μM +CBD
13	H_2O_2 50 μM +CBD

Figure S11. Western blot images of BJ-5ta, p 24. Original Western blot showing the COL3A1 bands. Blue arrows indicate bands used in Figure 7.

Deleted: 8

Formatted: Check spelling and grammar, Highlight

Deleted: 8

Formatted: Highlight

Elastin 70 kDa

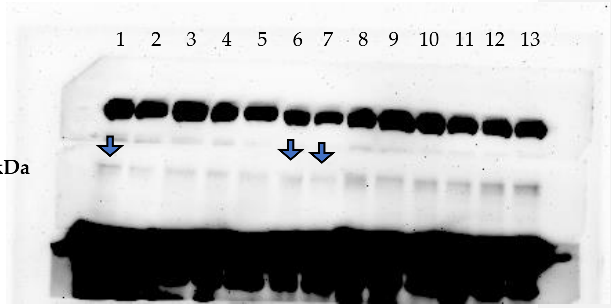

#	Sample
1	Untreated
2	PBS
3	DMSO
4	THC 2.0 μM
5	CBD 2.0 μM
6	H_2O_2 25 μM
7	H_2O_2 50 μM
8	H_2O_2 25 μM +DMSO
9	H_2O_2 50 μM +DMSO
10	H_2O_2 25 μM +THC
11	H_2O_2 50 μM +THC
12	H_2O_2 25 μM +CBD
13	H_2O_2 50 μM +CBD

Figure S12. Western blot images of BJ-5ta, p. 24. Original Western blot showing the Elastin bands. Blue arrows indicate bands used in Figure 7.

Deleted: 9

Formatted: Check spelling and grammar, Highlight

Deleted: 8

Formatted: Highlight

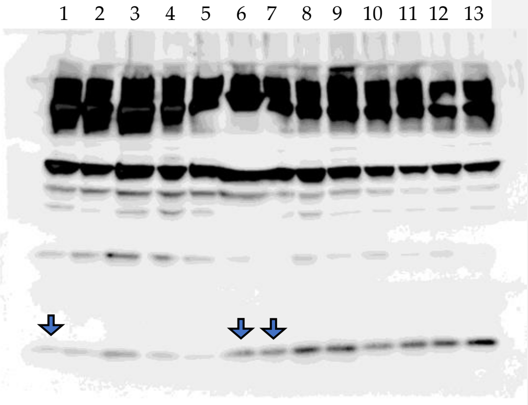

#	Sample
1	Untreated
2	PBS
3	DMSO
4	THC 2.0 μM
5	CBD 2.0 μM
6	H_2O_2 25 μM
7	H_2O_2 50 μM
8	H_2O_2 25 μM +DMSO
9	H_2O_2 50 μM +DMSO
10	H_2O_2 25 μM +THC
11	H_2O_2 50 μM +THC
12	H_2O_2 25 μM +CBD
13	H_2O_2 50 μM +CBD

Figure S13. Western blot images of BJ-5ta, p.24. Original Western blot showing the p16 bands. Blue arrows indicate bands used in Figure 7.

Deleted: 0
Formatted: Check spelling and grammar, Highlight
Deleted: 8
Formatted: Highlight

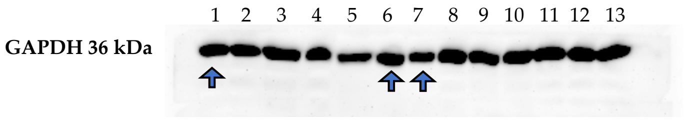

#	Sample
1	Untreated
2	PBS
3	DMSO
4	THC 2.0 μM
5	CBD 2.0 μM
6	H_2O_2 25 μM
7	H_2O_2 50 μM
8	H_2O_2 25 μM +DMSO
9	H_2O_2 50 μM +DMSO
10	H_2O_2 25 μM +THC
11	H_2O_2 50 μM +THC
12	H_2O_2 25 μM +CBD
13	H_2O_2 50 μM +CBD

Figure S14. Western blot images of BJ-5ta, p.24. Original Western blot showing the GAPDH bands. Blue arrows indicate bands used in Figure 7.

Deleted: 1

Formatted: Check spelling and grammar, Highlight

Deleted: 8

Formatted: Highlight

p21 21 kDa 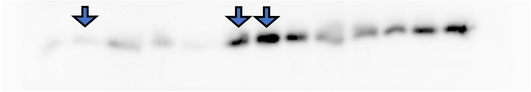

1 2 3 4 5 6 7 8 9 10 11 12 13

#	Sample
1	Untreated
2	PBS
3	DMSO
4	THC 2.0 μM
5	CBD 2.0 μM
6	H_2O_2 25 μM
7	H_2O_2 50 μM
8	H_2O_2 25 μM +DMSO
9	H_2O_2 50 μM +DMSO
10	H_2O_2 25 μM +THC
11	H_2O_2 50 μM +THC
12	H_2O_2 25 μM +CBD
13	H_2O_2 50 μM +CBD

Figure S15. Western blot images of BJ-5ta, p.24. Original Western blots showing the p21 bands. Blue arrows indicate bands used in Figure 7.

Deleted: 2

Formatted: Highlight

Deleted: 8

Formatted: Highlight

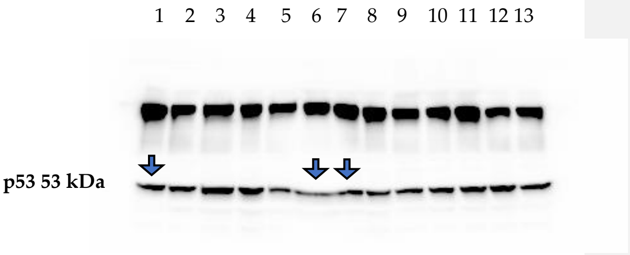

#	Sample
1	Untreated
2	PBS
3	DMSO
4	THC 2.0 μM
5	CBD 2.0 μM
6	H_2O_2 25 μM
7	H_2O_2 50 μM
8	H_2O_2 25 μM +DMSO
9	H_2O_2 50 μM +DMSO
10	H_2O_2 25 μM +THC
11	H_2O_2 50 μM +THC
12	H_2O_2 25 μM +CBD
13	H_2O_2 50 μM +CBD

Figure S16. Western blot images of BJ-5ta, p 24. Original Western blots showing the p53 bands. Blue arrows indicate bands used in Figure 7.

Deleted: 3

Formatted: Highlight

Deleted: 8

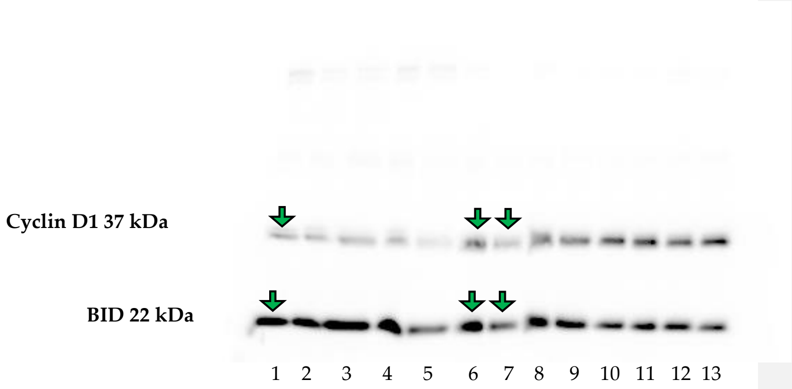

#	Sample
1	Untreated
2	PBS
3	DMSO
4	THC 2.0 μM
5	CBD 2.0 μM
6	H_2O_2 25 μM
7	H_2O_2 50 μM
8	H_2O_2 25 μM +DMSO
9	H_2O_2 50 μM +DMSO
10	H_2O_2 25 μM +THC
11	H_2O_2 50 μM +THC
12	H_2O_2 25 μM +CBD
13	H_2O_2 50 μM +CBD

Figure S17. Western blot images of BJ-5ta, p.24. Original Western blots showing the Cyclin D1 and BID bands. Green arrows indicate bands used in Figure 10.

Deleted: 4

Formatted: Highlight

Deleted: 11

Deleted: 0

GAPDH 36 kDa

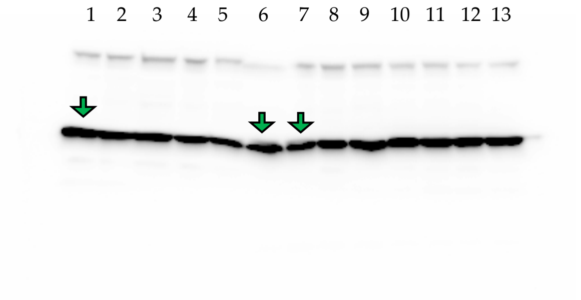

#	Sample
1	Untreated
2	PBS
3	DMSO
4	THC 2.0 μM
5	CBD 2.0 μM
6	H_2O_2 25 μM
7	H_2O_2 50 μM
8	H_2O_2 25 μM +DMSO
9	H_2O_2 50 μM +DMSO
10	H_2O_2 25 μM +THC
11	H_2O_2 50 μM +THC
12	H_2O_2 25 μM +CBD
13	H_2O_2 50 μM +CBD

Figure S18. Western blot images of BJ-5ta, p.24. Original Western blots showing the GAPDH bands. Green arrows indicate bands used in Figure 10.

Deleted: 5

Formatted: Highlight

Deleted: 11

Deleted: 0

Formatted: Highlight

SIRT1 120 kDa

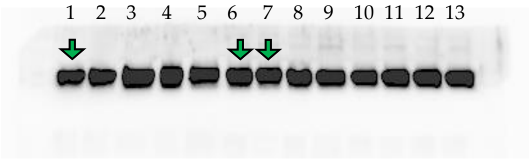

#	Sample
1	Untreated
2	PBS
3	DMSO
4	THC 2.0 μM
5	CBD 2.0 μM
6	H_2O_2 25 μM
7	H_2O_2 50 μM
8	H_2O_2 25 μM +DMSO
9	H_2O_2 50 μM +DMSO
10	H_2O_2 25 μM +THC
11	H_2O_2 50 μM +THC
12	H_2O_2 25 μM +CBD
13	H_2O_2 50 μM +CBD

Figure S19. Western blot images of BJ-5ta, p.24. Original Western blots showing the SIRT1 bands Green arrows indicate bands used in Figure 10.

Deleted: 6

Formatted: Highlight

Deleted: 11

Deleted: 0

Formatted: Highlight

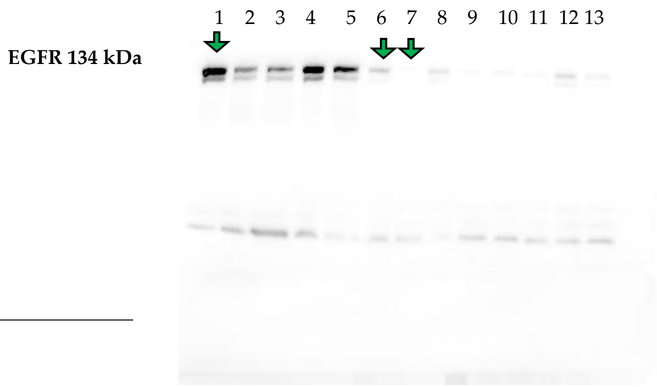

#	Sample
1	Untreated
2	PBS
3	DMSO
4	THC 2.0 μM
5	CBD 2.0 μM
6	H_2O_2 25 μM
7	H_2O_2 50 μM
8	H_2O_2 25 μM +DMSO
9	H_2O_2 50 μM +DMSO
10	H_2O_2 25 μM +THC
11	H_2O_2 50 μM +THC
12	H_2O_2 25 μM +CBD
13	H_2O_2 50 μM +CBD

Figure S20. Western blot images of BJ-5ta, p.24. Original Western blots showing the EGFR bands. Green arrows indicate bands used in Figure 10.

Deleted: 17
Formatted: Highlight
Deleted: 11
Deleted: 0
Formatted: Highlight

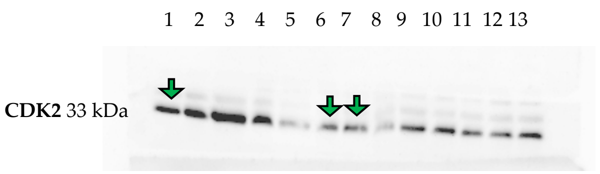

#	Sample
1	Untreated
2	PBS
3	DMSO
4	THC 2.0 μM
5	CBD 2.0 μM
6	H_2O_2 25 μM
7	H_2O_2 50 μM
8	H_2O_2 25 μM +DMSO
9	H_2O_2 50 μM +DMSO
10	H_2O_2 25 μM +THC
11	H_2O_2 50 μM +THC
12	H_2O_2 25 μM +CBD
13	H_2O_2 50 μM +CBD

Figure S21. Western blot images of BJ-5ta, p.24. Original Western blots showing the CDK2 bands. Green arrows indicate bands used in Figure 10.

Deleted: 18

Formatted: Highlight

Deleted: 11

Deleted: 3

c-Jun 21 kDa

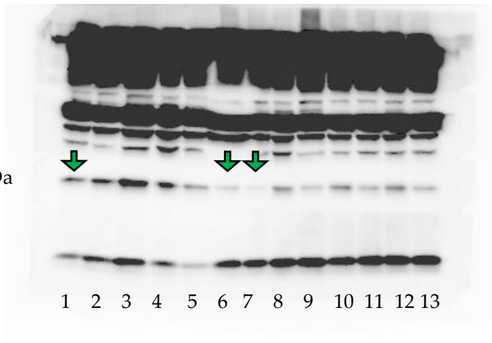

#	Sample
1	Untreated
2	PBS
3	DMSO
4	THC 2.0 μM
5	CBD 2.0 μM
6	H_2O_2 25 μM
7	H_2O_2 50 μM
8	H_2O_2 25 μM +DMSO
9	H_2O_2 50 μM +DMSO
10	H_2O_2 25 μM +THC
11	H_2O_2 50 μM +THC
12	H_2O_2 25 μM +CBD
13	H_2O_2 50 μM +CBD

Figure S22. Western blot images of BJ-5ta, p.24. Original Western blots showing the c-Jun bands. Green arrows indicate bands used in Figure 10.

Deleted: 19

Deleted: 11

Deleted: 3

Vinculin 124 kDa

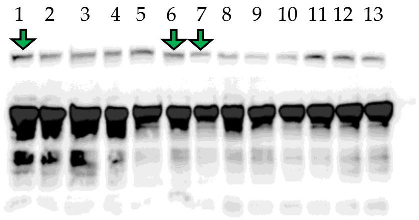

Membrane ½ -- over exposed. p.13/14

#	Sample
1	Untreated
2	PBS
3	DMSO
4	THC 2.0 μM
5	CBD 2.0 μM
6	H_2O_2 25 μM
7	H_2O_2 50 μM
8	H_2O_2 25 μM +DMSO
9	H_2O_2 50 μM +DMSO
10	H_2O_2 25 μM +THC
11	H_2O_2 50 μM +THC
12	H_2O_2 25 μM +CBD
13	H_2O_2 50 μM +CBD

Figure S23. Western blot images of BJ-5ta, p.24. Original Western blots showing the Vinculin bands. Green arrows indicate bands used in Figure 10.

Deleted: 0

Formatted: Highlight

Deleted: 11

Deleted: 3

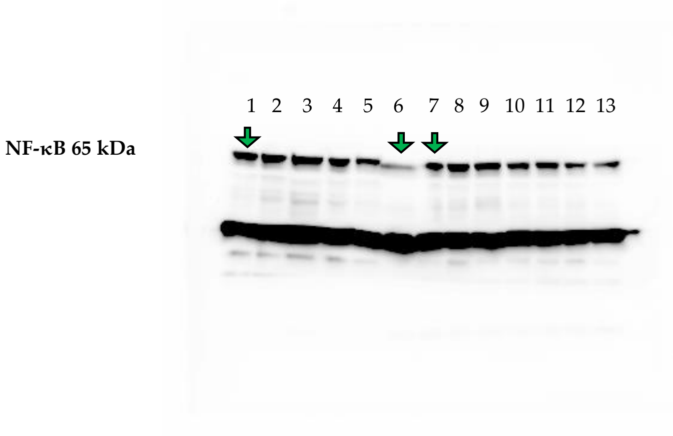

#	Sample
1	Untreated
2	PBS
3	DMSO
4	THC 2.0 μM
5	CBD 2.0 μM
6	H_2O_2 25 μM
7	H_2O_2 50 μM
8	H_2O_2 25 μM +DMSO
9	H_2O_2 50 μM +DMSO
10	H_2O_2 25 μM +THC
11	H_2O_2 50 μM +THC
12	H_2O_2 25 μM +CBD
13	H_2O_2 50 μM +CBD

Figure S24. Western blot images of BJ-5ta, p.24. Original Western blots showing the NF-κB bands. Green arrows indicate bands used in Figure 10.

Deleted: 1

Formatted: Highlight

Deleted: 11

Deleted: 3

Formatted: Check spelling and grammar, Highlight

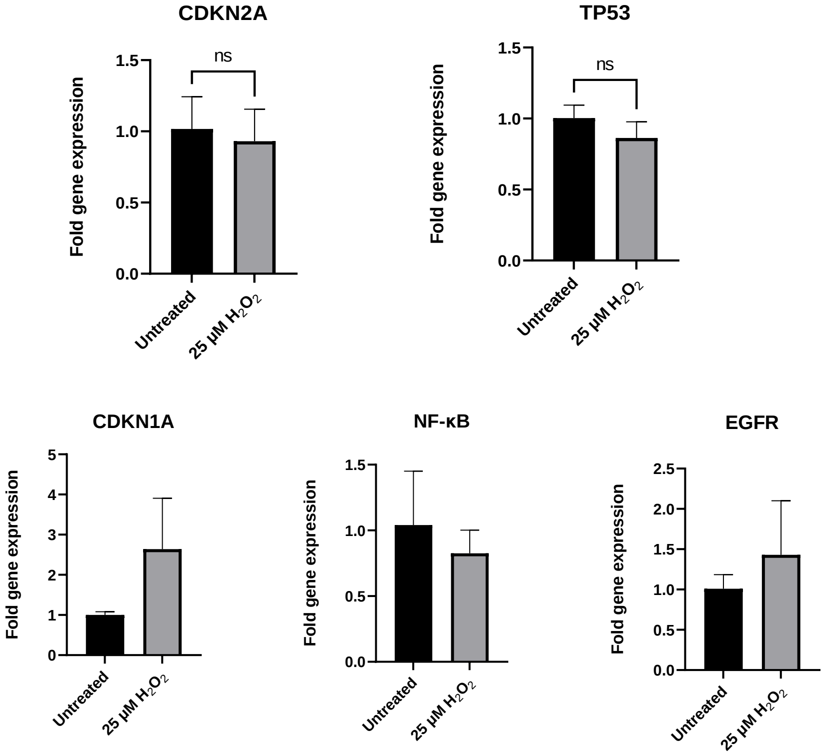

Figure S25. The effects of age on mRNA production of cell cycle checkpoint regulators in BJ-5ta (p.24) fibroblasts. Changes of mRNA expression as measured by RT-qPCR for *CDKN2A*, *TP53*, *CDKN1A*, *NF-κB*, and *EGFR* (from top left to bottom right, respectively). Data were analyzed with an unpaired Student's t-test. Significance (p) was indicated within the figures using the following scale: ns, not significant. Bars represent mean ± SD.

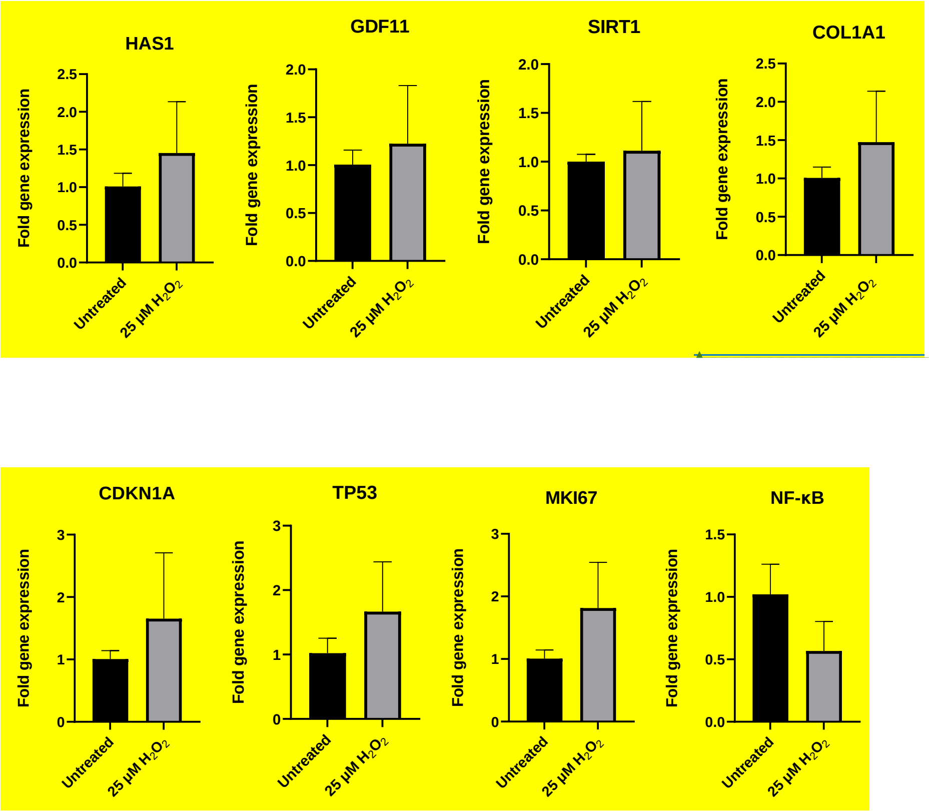

Figure S26. The effects of age on mRNA production of cell cycle checkpoint regulators in CCD-1064Sk (p.11) fibroblasts. Changes of mRNA expression as measured by RT-qPCR for *HAS1*, *GDF11*, *SIRT1*, *COL1A1*, *CDKN1A*, *TP53*, *MKI67*, *NF-κB* (from top left to bottom right, respectively). Data were analyzed with an unpaired Student's t-test. Significance (p) was indicated within the figures using the following scale: ns, not significant. Bars represent mean ± SD.

Field Code Changed

Table S1. Antibodies used for Western blots

Antibody	Supplier, Cat No	Dilution
Mouse anti-NFκB p65	Santa Cruz, sc-8008	1:500 in 5% milk (PBST)
Mouse anti-COL1A (COL-1)	Santa Cruz, sc-59772	1:100 in 5% milk (PBST)
Mouse anti-COL3A1 (B-10)	Santa Cruz, sc-271249	1:100 in 5% milk (PBST)
Mouse anti-elastin (BA-4)	Santa Cruz, sc-58756	1:200 in 5% milk (PBST)
Mouse anti-SIRT1 (B-7)	Santa Cruz, sc-74465	1:100 in 5% milk (PBST)
Mouse anti-CDKN2A/p16INK4a (F-12)	Santa Cruz, sc-1661	1:500 in 5% milk (PBST)
Mouse anti-p21 Waf1/Cip1/CDKN1A (F-5)	Santa Cruz, sc-6246	1:500 in 5% milk (PBST)
Mouse anti-cyclin D1 (DCS-6)	Santa Cruz, sc-20044	1:500 in 5% milk (PBST)
Mouse anti-CDK2	Santa Cruz, sc6248	1:500 in 5% milk (PBST)
Mouse anti-c-Jun (G-4)	Santa Cruz, sc-74543	1:200 in 5% milk (PBST)
Mouse anti-BID (E-7)	Santa Cruz, sc-514622	1:200 in 5% milk (PBST)
Mouse anti-EGFR (A-10)	Santa Cruz, sc-373746	1:200 in 5% milk (PBST)
Mouse anti-vinculin (7F9)	Santa Cruz, sc-73614	1:500 in 5% milk (PBST)
Mouse anti-p53 (DO-1)	Santa Cruz, sc-126	1:500 in 5% milk (PBST)
Mouse anti-GAPDH (0411)	Santa Cruz, sc-47724	1:1000 in 5% milk (PBST)
Bovine anti-Mouse	Santa Cruz, sc-2371	1:10000 in 5% milk (PBST)
Donkey anti-Rabbit	Santa Cruz, sc-2313	1:10000 in 5% milk (PBST)

Abcam, Abcam Inc, Cambridge, United Kingdom; **BSA**, Bovine Serum Albumin; **PBST**, 1x Phosphate-Buffered Saline, 0.1 % Tween® 20; **Santa Cruz**, Santa Cruz Biotechnology, Inc., Texas, United States; **Cell Signaling**, Cell Signaling Technologies, Massachusetts, United States

Table S2. Primer sequences for qPCR analysis

Target Gene	Sequence Forward (5' → 3')	Sequence Reverse (5' → 3')
COL1A1	CCACGACAAAGCAGAAACATC	GCAACACAGTTACACAAGGAAC
COL3A1	CTGGCATTCCTTCGACTTCT	AGCTTCAGGGCCTTCTTTAC
GDF11	TCTCAGAGCTAGTGTGGTAGAA	CCTCCCGGATCACTTTCAATAG
ELN	CTCAAAGCTGGATTCGCTCTA	AAGGGCAAGGTGGCTATTC
MMP1	CAGAAAGAGACAGGAGACATGAG	GAAGAGTTATCCCTTGCCTATCC
MMP2	AGAGAACCTCAGGGAGAGTAAG	CCTCGAACAGATGCCACAATA
HAS1	GTCTCCAGGGAGGGTATTTATTG	TCCTGATCACACAGTAGAAATGG
CDKN2A (P16)	AGCTGTCGACTTCATGACAAG	GAGCTTTGGTTCTGCCATTTG
EGFR	CAAGGAAGCCAAGCCAAATG	CCGTGGTCATGCTCCAATAA
MKI67	GGAGCCAGGTGACATCATAAA	CATGGATGACGCTGTGAGAA
CDKN1A (P21)	CCTTCCAGCTCCTGTAACATAC	TCGAGAGGTTTACAGTCTAGGT
SIRT1	AGAACCCATGGAGGATGAAAG	TCATCTCCATCAGTCCCAAATC
SIRT3	CCTCCTTCCTAGCATCACATTAC	CCTGGGAGTCACTGTCATTAAA
SIRT4	GAACCTGGAACAGGGACTTT	CTTTGTCAGTGCACCCTACT
SIRT6	CCTCTGACTTGCTGTGTTGT	GAGGGAGTTCACTCCTGTTTAAG
TP53	AGGGATGTTTGGGAGATGTAAG	CCTGGTTAGTACGGTGAAGTG
NFKB1	GAGACATCCTTCCGCAAACT	GGTCCTTCCTGCCCATAATC
TIMP1	TCCCAGATAGCCTGAATCCT	TGCTGGGTGGTAACTCTTTATT
GAPDH	CAGGAGGCATTGCTGATGAT	GAAGGCTGGGGCTCATTT
